# Multi-modal single-cell sequencing reveals network transition in circulating monocytes that aligns with faster recovery in patients with trauma and favours a response to M-CSF

**DOI:** 10.1016/j.ebiom.2026.106368

**Published:** 2026-07-03

**Authors:** Tianmeng Chen, Julia Hughes, Julia Cornoy, Wei Chen, Richard Duerr, Timothy Billiar

**Affiliations:** a Department of Surgery, University of Pittsburgh, Pittsburgh, PA, 15213, USA; b Department of Medicine, University of Pittsburgh, Pittsburgh, PA, 15213, USA; c Department of Pediatrics, University of Pittsburgh, Pittsburgh, PA, 15224, USA

**Keywords:** DOGMA-seq, scRNA-seq, Trauma, Monocytes, Macrophages

## Abstract

**Background:**

Previous transcriptomic studies demonstrated an acute genomic storm in circulating immune cells after trauma that gradually returns to baseline. The magnitude and duration of these changes is associated with post-injury complications. We hypothesised that other immune cell features emerging in the subacute timeframe should be associated with recovery.

**Methods:**

We applied DOGMA-seq on peripheral blood mononuclear cells isolated at day 3 after injury from the patients undergoing slow or fast recovery from critical illness (n = 8/group), along with age-and-sex matched healthy controls. We explored the functional responses of newly identified gene co-expression networks using *in vitro* cell culture (GM-CSF or M-CSF induced macrophage differentiation) followed by scRNA-seq.

**Findings:**

We identified a subset of CD172a hi/MHCII hi, CD14+ monocytes that were distinct from baseline, and overrepresented in patients that recovered faster and associated with changes in several key gene co-expression networks. A gene co-expression pattern associated with chemotaxis and cell adhesion was overrepresented in patients with fast recovery and, specifically associated with increased accessibility of AP1 family motifs, continuously deviating from baseline, and favouring a response to M-CSF rather than GM-CSF during macrophage differentiation.

**Interpretation:**

Our findings add a new information layer to the current paradigm by demonstrating that recovery is not simply a return to the baseline state and instead involves the emergence of new monocyte subset with new transcriptomic programs that may influence the macrophage response.

**Funding:**

National Institutes of Health R35 grant R35GM127027 (T.B.).

**Research in context**

**Evidence before this study**

Trauma is the leading cause of death for individuals under 45 years old, and the third leading cause of death overall. A dysregulated immune response is regarded as a major contributor to post-injury complications and mortality. The long-standing paradigm defining the early immune response to trauma (Xiao et al. JEM 2011) includes a dramatic upregulation of inflammatory features and simultaneous downregulation of adaptive immune signalling pathways within minutes to hours after severe injury. These changes gradually return to baseline and the magnitude and duration of these patterns associate with complicated recoveries. This paradigm is primarily comprised of the changes initiated very early after injury. Whether other changes in immune cells that associate with clinical course emerge in the sub-acute time frame remains unclear.

DOGMA-seq simultaneously measures transcriptomic changes, open chromatin patterns and surface epitope expression in the same cells, which provides an opportunity to deconvolute cellular immune responses at high resolution across multiple data layers. A PubMed search using “(DOGMA-seq) AND (trauma)” identified no DOGMA-seq studies in human trauma.

**Added value of this study**

In this study, we go well beyond the current paradigm to demonstrate that patient recovery after severe injury is not simply a return of immune cell states to baseline. Instead, a new subset of CD14+ monocytes emerges in the circulation in the sub-acute time frame in patients destined for a faster recovery from critical illness. These cells are characterised by an upregulation of surface CD172a and a return of MHCII expression; changes that are distinct from steady state CD14+ monocytes. The relative abundance of these resolving monocytes increases over time and with patient recovery. The unique features of these cells include distinct gene co-expression networks (green-shifted pattern) and open chromatin patterns (increased accessibility of AP1 family motifs). Furthermore, during macrophage differentiation, the cells with a green-shifted pattern exhibited a higher response to M-CSF, while the response to GM-CSF remained the same.

**Implications of all the available evidence**

Based on our new findings in combination with previous observations, we proposed a model that adds a third dimension to the existing paradigm for the early immune response to trauma. Not only do the pro-inflammatory and immune suppression patterns in circulating monocytes return to baseline, but a monocyte subset emerges that is primed to respond to specific cytokine signals (e.g., M-CSF) to migrate into tissues. Because macrophages in tissue can persist for long periods, this network transition in circulating monocytes could have a profound influence on tissue immune responses throughout the body. Further investigation on how to modulate this network transition could provide strategies to reshape the immune response in peripheral tissues and improve patient outcomes after severe injury.

## Introduction

Trauma, both accidental and intentional, is the leading cause of death for individuals under 45 years old, and the third leading cause of death overall.^1^ A dysregulated immune response is regarded as a major contributor to post-injury complications and mortality.^2^ In humans, the cellular changes of this systemic response have been assessed by bulk transcriptomics of whole blood leukocytes,^3^^,^^4^ flow cytometry of isolated immune cell populations,^5^ and single-cell studies of peripheral blood mononuclear cells (PMBCs) including CyTOF (cytometry by time-of-flight),^6^ transcriptomics,^7^ and open chromatin patterns.^8^ These studies have informed the prevailing paradigm that involves a near immediate induction of proinflammatory innate immune responses and simultaneous suppression of adaptive immunity genes; responses that are excessive and sustained in patients with complicated recoveries.^3^

Advances in single cell sequencing technologies have provided new insights into the heterogeneity of immune cell populations in different diseases.^9^ Our previous single-cell transcriptomic analysis (scRNA-seq) of PBMCs from severely injured patients established that the major early changes in circulating immune cells took place in CD14+ monocytes and neutrophils.^7^ A transcriptomic signature derived from CD14+ monocytes within 4 h of injury captured the major immune cell changes including, an upregulation of proinflammatory genes with the simultaneous suppression of HLA (Human Leucocyte Antigen) class II and interferon signalling genes. This signature classified patients into two signature groups (SG subtypes) associated with different clinical prognosis.^7^ A separate single-cell Assay for Transposase-Accessible Chromatin sequencing (scATAC-seq) analysis of PBMC identified unexpected global epigenetic alterations in circulating leukocytes that were present upon admission after severe injury. The genes associated with the global epigenetic features were used to define epigenetic patient subtypes (EG subtypes) that aligned with different outcomes independently of the SG subtypes.^8^ The biology identified in these studies was present within hours of severe injury and therefore represents the features of the initial systemic immune response to severe injury. However, whether immune cells associated with early recovery would emerge in the sub-acute time frame remains unclear.

To assess divergence in immune cell subsets based on clinical trajectory, we carried out trimodal single cell analysis on PBMCs isolated from severely injured humans at 72 h after polytrauma and admitted to the intensive care unit with organ dysfunction. This single cell method known as DOGMA-seq^10^ simultaneously measures transcriptomic changes, open chromatin patterns and surface epitope expression in the same cells. By sequencing samples from the patients that recovered from critical illness early (ICU length of stay [LOS] less than 7 days) and those that did not, we identified a monocyte subset that appeared days prior to recovery and that increased in prevalence along the recovery trajectory. Importantly, these cells expressed a combination of transcriptomic, epigenetic and surface markers not previously described in CD14+ monocytes and were not cells simply transitioning back to a baseline state. Gene co-expression network analysis identified the corresponding gene network transition that included enhanced chemotaxis and cell adhesion, recovered HLA class II expression and subsided inflammation; findings that were validated in an external transcriptomic dataset from a large cohort of patients with trauma. *In vitro* cytokine stimulation studies established that this network transition favoured a higher response to M-CSF (Macrophage Colony-Stimulating Factor) for macrophage differentiation, without obviously affecting the response to GM-CSF (Granulocyte-Macrophage Colony-Stimulating Factor). These findings refine the paradigm for the early human immune response to severe injury by revealing the gene network transition within CD14+ monocytes initiated prior to clinical resolution of critical illness; changes that may re-shape the innate and adaptive immune responses within tissues via differentiated macrophages.

## Methods

### Study design and participants

Patients with evidence of polytrauma and the presence of shock on presentation (systolic BP < 90 mmHg and heart rate >108 beats/min) with subsequent admission to the intensive care unit of UPMC Presbyterian University Hospital were eligible for enrolment. Blood samples were obtained at day 3 and day 7 after injury for PBMC isolation and cryopreserved in liquid nitrogen using an existing biobank. Sixteen patients (8 FR and 8 SR) and four healthy controls (HCs) were selected for DOGMA-seq analysis. FR was defined as ICU LOS (length of stay) ≤ 7 days and SR as ICU LOS >7 days. Patients with isolated traumatic brain injury were excluded. Patient characteristics and batch design are shown in Tables 1 and 2. Age and sex were balanced between FR and SR within each batch.

**Table 1:** Single-cell datasets generated in this study.

Dataset name	Sequencing	Cell type	Participants	Time points	Comparison
DOGMA-seq	DOGMA-seq	PBMCs after thawing	8 Patients with SR + 8 Patients with FR + 4 healthy participants (HC)	D3	HC vs. FR vs. SR
D3D7	scRNA-seq	PBMCs after thawing, flow deleted lymphocytes	2 Patients with SR + 2 Patients with FR	D3, D7	1 FR vs. SR 2 D3 vs. D7
Ma-trauma	scRNA-seq	PBMCs from patients with trauma cultured in different conditions, flow enriched for CD11b+ cells	3 patients with trauma	D3, D7	1 Ctrl vs. GM-CSF vs. M-CSF 2 D3 vs. D7
Ma-healthy	scRNA-seq	PBMCs from healthy participants cultured in different conditions, flow enriched for CD11b+ cells	2 healthy participants	N/A	1 Ctrl vs. GM-CSF vs. M-CSF 2 D3 vs. D7

**Table 2:** Sample information for DOGMA-seq dataset.

Sample ID	Batch^a^	age	gender	Injury	ICU days
MM4018	P1	25	Male	Blunt	21
MM4023	P1	24	Male	Blunt	4
MM4039	P1	33	Male	Blunt	44
MM4056	P1	20	Male	Penetrating	3
MM4012	P2b	45	Female	Blunt	11
MM4015	P2b	21	Female	Blunt	1
MM4017	P2b	41	Female	Penetrating	4
MM4026	P2b	42	Male	Blunt	6
MM4043	P2b	21	Female	Blunt	17
MM4052	P2b	41	Male	Blunt	13
HC3527	P2b	37	Female	N/A	HC
HC3508	P2b	35	Male	N/A	HC
MM4004	P3a, P3b	77	Female	Blunt	6
MM4006	P3a, P3b	60	Male	Blunt	3
MM4008	P3a, P3b	33	Female	Blunt	5
MM4014	P3a, P3b	63	Male	Blunt	22
MM4027	P3a, P3b	27	Female	Blunt	15
MM4040	P3a, P3b	73	Female	Blunt	26
HC3522	P3a, P3b	64	Female	N/A	HC
HC3520	P3a, P3b	63	Male	N/A	HC

a a, b are technical replicates, which is the same cell suspension loaded on two different wells on the 10x chip. P2a QC was low and excluded.

### Ethics

Patients with trauma and healthy volunteers were enrolled in an observational study approved by the University of Pittsburgh Institutional Review Board (IRB protocol number: 19040329). Informed consent was obtained from all the subjects or next of kin.

### Statistics

Establishing an appropriate sample size for this study was empirical; however, to our knowledge, this study represents one of the largest datasets generated using DOGMA-seq in human PBMCs. Based on empirical estimates from a previous study using a similar experimental setup,^11^ this scale of analysis enables the detection of an adequate number of differentially expressed genes (DEGs) between groups. Therefore, we assert that the current sample size is sufficient to identify biologically meaningful and statistically significant differences. In addition, the cell subsets and transcriptional signatures derived from the DOGMA-seq data were independently validated using flow cytometry as well as both published and newly generated datasets, further supporting the robustness and reproducibility of our conclusions.

### Justification for day 3 timepoint selection

It is known that organ dysfunction peaks in the first 2–3 days after severe injury and that patients then diverge into those with slow resolution of organ dysfunction and those with a faster resolution.^4^ In addition, the Glue Grant Genomic Storm paper published by Xiao et al.^3^ divided the patient outcomes into uncomplicated (time-to-recovery TTR <5 days), intermediate (TTR = 5–14 days), complicated (TTR > 14 days). Thus, the factors that determine different recovery trajectories, if they exist, should be present within 5 days after injury. These observations provide the rationale for choosing day 3 in this study.

### PBMC cryopreservation and thawing

Blood samples were obtained from all study participants. PBMCs were isolated by standard Ficoll centrifugation. The isolated cells were cryopreserved. The 4 samples from 4 different participants were thawed to generate this dataset. The 4 samples were processed in parallel, which were thawed in the 37 °C water bath, and transferred to a 50 mL conical tube after thawing was complete. As suggested by 10x Genomics protocol,^12^ 1 mL of thawing medium (RPMI with 10% FBS) was added dropwise (5 sec/drop), followed by 2 mL, 4 mL, 8 mL, 16 mL thawing medium at ∼ 1-min intervals.

### Flow cytometry and sorting

Cells were washed with PBS once, and incubated with Fc block for 10 min, then stained with antibodies and Live/Dead dye in PBS + 2%FBS (FACS buffer) on ice for 30 min. After one wash of FACS buffer, cells were finally resuspended in FACS buffer. Single-stained compensation beads and unstained cells were used to set up voltages and compensation parameters. Flow cytometry was performed using BD LSRII flow cytometer (BD Biosciences, Becton, Dickinson and Company). Flow sorting was performed using Aria 5 lasers flow sorter. Data was analysed using FlowJo v10.0.7.

Human TruStain FcX (Fc receptor blocking solution): BioLegend, Cat# 422302, RRID:AB_2818986.

Live/Dead Aqua Dead Cell Stain dye: Thermo Fisher Scientific, Cat# L34966.

UltraComp eBead Plus Compensation Beads: Thermo Fisher Scientific, Cat# 01-3333-42.

Pacific Blue anti-human CD11b Antibody (clone: ICRF44): BioLegend, Cat# 301315, RRID:AB_493015.

APC/Fire 750 anti-human HLA-DR, DP, DQ Antibody (clone: Tü39): BioLegend, Cat# 361712, RRID:AB_2750314.

APC anti-human CD172a (SIRPα) Antibody (clone: 15–414): BioLegend, # 372106, RRID:AB_2650863.

PE-Cy7 anti-human CD14 Antibody (clone: HCD14): BioLegend, Cat# 325617, RRID:AB_830690.

Super bright 600 anti-human CD3 Antibody (clone: OKT3): Thermo Fisher Scientific, Cat# 63-0037-41, RRID:AB_2637435.

PE/Dazzle 594 anti-human CD19 Antibody (clone: HIB19): BioLegend, Cat# 302252, RRID:AB_2563560.

### DOGMA-seq library preparation

Eight samples were processed in each batch. Total 20 samples (16 patients with trauma and 4 HCs) were processed in three different batches. For each sample, ∼0.5 million cells were transferred into two 1.5 mL low-binding tubes, incubated with Human TruStain FcX™ (BioLegend) for 10 min and then incubated with a unique TotalSeq™-A anti-human Hashtag antibody (BioLegend) for 30 min. After 3 times of wash (1350 rpm ∗ 5 min, PBS + 2%FBS), 187,500 cells/tube were pooled into one 1.5 mL low-binding tube and incubated with TotalSeq™-A Human Universal Cocktail, V1.0 (BioLegend) for 30 min. After 3 times of wash (1350 rpm ∗ 5 min, PBS + 2%FBS), cells were permeabilised with 100 ul Digitonin lysis buffer for 1 min, and then washed with 1 mL Digitonin wash buffer (pipette 5 times up and down, then 1350 rpm ∗ 5 min). All the reactions were performed at 4 °C. Finally, 30,000 nucleus were loaded on one well of 10x Genomics Chip (2 wells for 8 samples). Chromium Next GEM Single Cell Multiome ATAC + Gene Expression Reagent Kits were purchased to prepare GEX and ATAC libraries. All the remaining steps of DOGMA-seq library preparation were the same as previously described.^10^

TotalSeq™-A0251 anti-human Hashtag 1 Antibody: BioLegend, Cat# 394601, RRID:AB_2750015.

TotalSeq™-A0252 anti-human Hashtag 2 Antibody: BioLegend, Cat# 394603, RRID:AB_2750016.

TotalSeq™-A0253 anti-human Hashtag 3 Antibody: BioLegend, Cat# 394605, RRID:AB_2750017.

TotalSeq™-A0254 anti-human Hashtag 4 Antibody: BioLegend, Cat# 394607, RRID:AB_2750018.

TotalSeq™-A0255 anti-human Hashtag 5 Antibody: BioLegend, Cat# 394609, RRID:AB_2750019.

TotalSeq™-A0256 anti-human Hashtag 6 Antibody: BioLegend, Cat# 394611, RRID:AB_2750020.

TotalSeq™-A0257 anti-human Hashtag 7 Antibody: BioLegend, Cat# 394613, RRID:AB_2750021.

TotalSeq™-A0258 anti-human Hashtag 8 Antibody: BioLegend, Cat# 394615, RRID:AB_2750022.

TotalSeq™-A Human Universal Cocktail, V1.0: BioLegend, Cat# 399907, RRID:AB_2888692.

### scRNA-seq library preparation

Flow isolated monocytes were further processed with scRNA libraries using Chromium Single Cell 3′ Reagent Kits (v3.1, CG000315) as previously described.^7^

### Single-cell library sequencing

Libraries were paird-end sequenced in UPMC (University of Pittsburgh Medical Center) Genomic Center/Pitt High Throughput Genomics Core, with estimated 50,000 reads per cell for GEX, 3000 reads per cell for HTO (Hashtag Oligonucleotide), 24,450 reads per cell for ADT (Antibody-Derived Tag), 50,000 reads per cell for ATAC.

### DOGMA-seq data analysis

For each sample, HTO and ADT fastq files were processed by kite (https://github.com/pachterlab/kite) to get an ADT count matrix and a HTO count matrix. For multiome data (GEX and ATAC), feature-barcode count matrix was generated using *cellranger-arc count* (mapped to GRCh38 human reference genome) and *cellranger-arc aggr* (cellranger-arc/2.0.0). Seurat^13^ and Signac^14^ were used to create an object for the feature-barcode count matrix of multiome data, and ADT matrix and HTO matrix were added to this Seurat object.

For each batch of data, cells were demultiplexed using Seurat (v4.0.5), and only singlets were kept for the downstream analysis. Data integration was performed to correct batch effects across different batches using Seurat. The three different layers of data (GEX, ATAC, ADT) were first integrated separately. For each data layer, single-modal data were normalised within each batch. The top variable features were identified, and the features that were repeatedly variable across donors were selected to perform data integration. After that, WNN (weighted-nearest neighbour) analysis was performed and cell clusters were identified using the first 30 PCs (Principal Component) from the integrated GEX data, the first 30 PCs from the integrated ADT data, and 2–30 LSIs (Latent Semantic Indexing) from the integrated ATAC data.

#### Identification of differential genes, surface markers and motifs

To identify differential genes or surface markers, we used unintegrated and normalised GEX or ADT data with donor as a potential variable. We computed motif activity score for each cell by running chromVAR,^15^ and differential motifs were identified between groups using computed activity score matrix with donor as a potential variable. Transcription factor binding profiles were obtained from JASPAR2020 R package (R version 4.1.0).^16^

#### Identification of PC associated genes

For a specific PC, Pearson's correlation test was performed between the normalised gene expression values (of a specific gene) and the PC1 coordinates across all single cells. The raw p value was corrected by the Benjamini-Hochberg method for multiple testing. The significant correlated genes (FDR < 0.001 and |r| > 0.1) were ranked by correlation coefficient and used as the input for GSEA.

#### Identification of gene clusters

The union of pairwise DEGs (|fold change| > 2) were included in this analysis. Gene-by-cluster pseudobulk matrix was generated by taking the average of the normalised gene expression values for each gene within each cell cluster. Hierarchical clustering was used to identify gene clusters. Ward clustering was performed using hclust() function in R with the agglomeration method set as “ward.D2”. The distance matrix was 1 minus Pearson's correlation.

#### Association between gene modules and motifs

For a specific gene module identified from hdWGCNA (i.e. red, green or pink module), signature score was calculated for each single cell using GEX data. Motif activity score for each motif for each cell was computed by chromVAR using ATAC data. Linear regression was fitted between the signature scores of each module and the activity scores of each motif across all single cells with different batches as a covariate. Raw p value was corrected by Benjamini-Hochberg method for multiple testing.

#### Mapping and annotating query dataset

We used the GEX data from the DOGMA-seq dataset as reference. The identified CD14+ monocyte cell clusters were transferred to the D3D7 dataset using Seurat package. We first identified a set of anchors between the reference and query datasets using FindTransferAnchors( function. Then, we projected the query onto the reference UMAP structure using MapQuery() function.

### scRNA-seq data analysis

scRNA-seq data analysis was the same as the GEX data analysis from DOGMA-seq data.

### Gene co-expression network analysis

We identified consensus co-expression networks (modules) using hdWGCNA (v0.4.8)^17^ in D3D7 dataset across four patients with trauma, following the tutorial of https://smorabit.github.io/hdWGCNA/articles/consensus_wgcna.html. A separate expression matrix was set up for each patient. The co-expression network was first constructed in each individual separately, and then the networks were integrated, and the gene modules were identified. The identified gene modules in D3D7 dataset were projected to the scRNA-seq data from DOGMA-seq dataset, following the tutorial of https://smorabit.github.io/hdWGCNA/articles/projecting_modules.html.

### Signature score calculation

For specific signature (a set of genes), signature score was calculated by averaging of the scaled gene expression values of these genes.

### Gene set enrichment analysis

For a pre-ranked gene list, GSEA^18^ was performed using the fgsea R package (v1.10.1). For the gene lists without rank, fold enrichment and hypergeometric p value were computed. Raw p value was corrected using Benjamini-Hochberg method for multiple testing.

### Time-to-event analysis

The bulk gene array datasets of patients with trauma (GSE36809)^3^ have multiple time points for each patient. The sampling time points were around 12 h, 1 d, 4 d, 7 d, 14 d, 21 d or 28 d according to the original paper. Thus, the actual time points were binned into the nearest time point mentioned above. Within each time bin, if a patient has >1 data points, only the first data point was included in the analysis. Only the data points before patients’ recovery were analysed. Event was set as recovery (0- unrecovered; 1- recovered). For the dead patients, “hospital length of stay” was used as the time and recovery status was annotated as “0”. Cox proportional hazards model was performed by coxph() function in R adjusting for covariates using survival package (v3.1.8). Kaplan–Meier curves were also generated for each time point and compared using the log-rank test. Patients were stratified into high- and low-risk groups based on the median signature score.

### Deconvolution of cell composition

The major cell composition in the bulk gene array datasets of patients with trauma (GSE36809)^3^ were deconvoluted using CIBERSORT.^19^ CEL files were downloaded and processed by CEL_to_mixture.R (provided by CIBERSORT) to prepare the input of gene expression matrix. LM22 matrix (provided by CIBERSORT, containing 22 functionally defined human immune subsets) was used as the input of signature matrix.

### Cell culture *in vitro*

On day 0, cryopreserved PBMCs were thawed and resuspended in complete media (RPMI164+ 10%FBS + penicillin (100U/mL) + streptomycin (100U/mL)) at 5 ∗ 10ˆ5 cell/mL at 37° with 5%CO2 overnight. On day 1, GM-CSF (Sigma–Aldrich, Cat# G5035; final concentration: 50 ng/mL) or M-CSF (Sigma–Aldrich, Cat# H6916; final concentration: 50 ng/mL) were added to the media. On day 4, the same amount of fresh complete media with or without cytokines correspondingly were added to the cultured cells. Cells were harvested on day 7 (Accutase: Sigma–Aldrich, Cat# A6964).

### Role of funders

The funders had no roles in study design, data collection, data analysis, data interpretation, or writing of the report.

## Results

### DOGMA-seq identifies a monocyte subset M_C3 dominant in FR

**Fig. 1:**
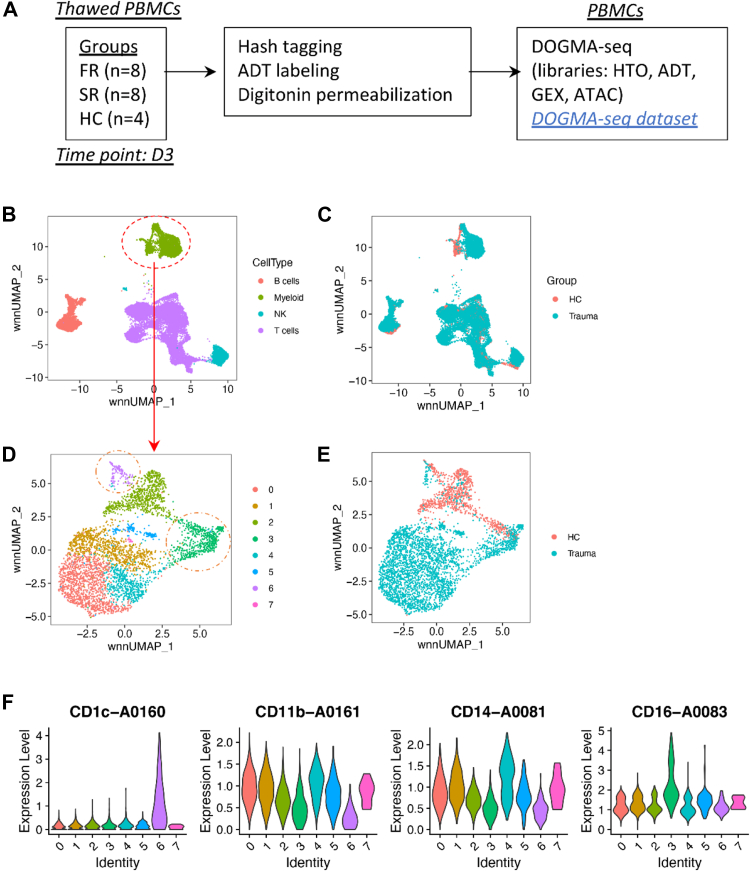
**Multimodal single-cell profile of PBMCs isolated from patients with trauma.** (A) Experimental design and workflow to generate the DOGMA-seq dataset. (B–C) The weighted nearest neighbour (wnn) UMAP plot shows all the PBMCs passed the quality control and are colour coded by major cell types (B) and groups (C). (D–F) Myeloid cells were extracted and analysed separately. wnnUMAP plot was colour coded by cell clusters (D) and groups (E). (F) ADT levels of representative surface markers are shown across different myeloid cell clusters.

To determine if clinical trajectory-associated immune cell subsets could be identified in the circulation of severely injured patients in the subacute time frame, we isolated PBMCs from patients with trauma on day (D) 3 from cohorts of patients undergoing either fast or slow recovery (FR vs. SR). Enrolment criteria included evidence of polytrauma and the presence of shock on presentation (systolic BP < 90 mmHg and heart rate >108 beats/min) with subsequent admission to the ICU. FR was defined as ICU LOS (length of stay) < 7 days and SR as ICU LOS >7 days. In the initial experiment, PBMC from 16 patients (8 FR and 8 SR, patient characteristics and batch design shown in Tables 1 and 2) and four HCs were subjected to trimodal single cell analysis (RNA, ATAC, and ADT assessment, Fig. 1A) known as DOGMA-seq.^10^ Within each batch, sex and age matched patients with SR and FR were included. Differences across batches were corrected by data integration. The libraries generated from this analysis, referred to here as the DOGMA-seq dataset, were used to compute UMAP and identify cell clusters based on the tri-model sequencing data. Fig. 1B shows the weighted nearest neighbour UMAPs (wnnUMAP) that integrate the data across all three modalities for all the PBMC cell populations (Fig. S1A–B). Consistent with our previous reports,^7^^,^^8^ there was obvious separation in cells from patients with trauma compared to those from HC, especially in monocytes (Fig. 1C). The remainder of this study provides an in-depth analysis of monocyte populations.

**Fig. 2:**
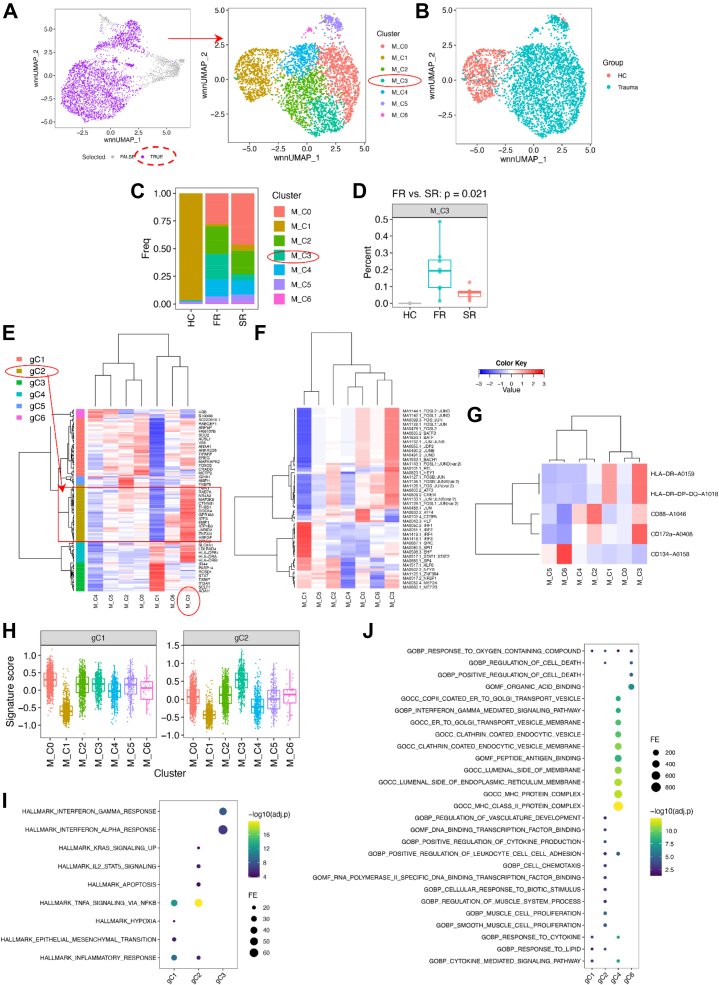
**DOGMA-seq identifies a CD14+ monocyte subset M_C3 dominant in patients with trauma with fast recovery.** (A) CD14+ monocytes were extracted and analysed separately. Seven clusters (M_C0–C6) were identified and shown in the wnnUMAP plot. (B) wnnUMAP plot colour coded by groups. (C) Cell cluster composition shown by patients undergoing different recovery trajectories. (D) The percentage of M_C3 clusters is shown by individuals. Each dot represents an individual. The differences between FR and SR were tested by Wilcoxon test. (E–G) The profile of GEX (E), ATAC (F) and ADT (G) across different cell clusters. Pairwise DEGs, motifs or ADTs were identified between each combination of two cell clusters. A pseudobulk matrix was generated by averaging the values within each cell cluster. Ward clustering was used to identify gene clusters (gC). The distance matrix was 1 minus Pearson's correlation. (H) Signature scores of gC1 and gC2 were calculated and are shown by different cell clusters. Each dot represents a single cell. (I–J) Results of gene set overrepresentation test are shown. An adjusted p value < 0.05 was defined as significant. Gene sets are ordered by fold enrichment (FE) scores. Top 10 gene sets are shown for each gC. (I) Significant hallmark gene sets. (J) Significant GO terms. The boxes shown in boxplot span from the Q1 to the Q3, with the centreline showing the median. Lower whiskers represent Q1 − 1.5∗IQR, and upper whiskers represent Q3 + 1.5∗IQR (Q1: the first quantile, Q3: the third quantile, IQR = Q3 − Q1).

Focussing next on only cells from the myeloid compartment, we found that CD16+ monocytes and DCs from patients with trauma and HCs fell into the same cluster. In contrast, CD14+ monocytes were largely separated between patients vs. HC, indicating that the major changes took place in this cell population (Fig. 1D–F). Within CD14+ monocytes (Fig. 2A), we identified seven cell clusters (M_C0–C6). M_C1 largely corresponded to cells from HC (Fig. 2B–C). Of the clusters associated with patients with trauma, M_C3 was the only cell cluster that achieved significant differences between patients with SR and FR (Wilcox test: p = 0.02) and was dominant in Patients with FR (Fig. 2C–D, and Fig. S2). To characterise the differences across cell clusters, we identified pairwise differentially expressed genes (DEGs) between each combination of two cell clusters, and these DEGs yielded six gene clusters (gC1–6, Fig. 2E). The HC-enriched cluster M_C1 had a high expression of HLA-II genes and very distinct gene expression profile from all the clusters associated with patients with trauma. It is notable that the multi-modal features of M_C3 indicate this cell subset was not simply transitioning back to a baseline monocyte state. HLA-II gene expression (gC4), which is known to be suppressed early after trauma,^2^^,^^3^^,^^7^ was comparable in M_C3 to HC (M_C1). However, M_C3 highly expressed a gene cluster (gC2) not seen in any other trauma-related clusters or the HC-related cluster (Fig. 2E). The ATAC profile shows that the accessibility of AP1 (Activator Protein 1) motifs (JUN/FOS) are upregulated after trauma and highest in M_C3 monocytes (Fig. 2F). ADT analysis showed that M_C3 highly expressed HLA-II and CD172a surface proteins (Fig. 2G), providing a potential means to quantify and enrich for these cells through flow cytometry. The signature scores of gC1 and gC2 across different monocyte clusters are shown in Fig. 2H. The gene cluster gC1 includes the genes that are generally upregulated across all monocytes after trauma, while gC2 contains the genes that are specifically upregulated in M_C3 monocytes after trauma. Gene set enrichment analysis indicated that both gC1 and gC2 are enriched in inflammatory pathways, as expected after severe trauma. However, gC2 is more associated with chemotaxis and cell adhesion (Fig. 2I–J). The upregulation of chemotaxis and adhesion genes suggested that the M_C3 monocytes may be programed to exit the circulation.

### scRNA-seq demonstrates a further increase in M_C3 along the recovery timeline

**Fig. 3:**
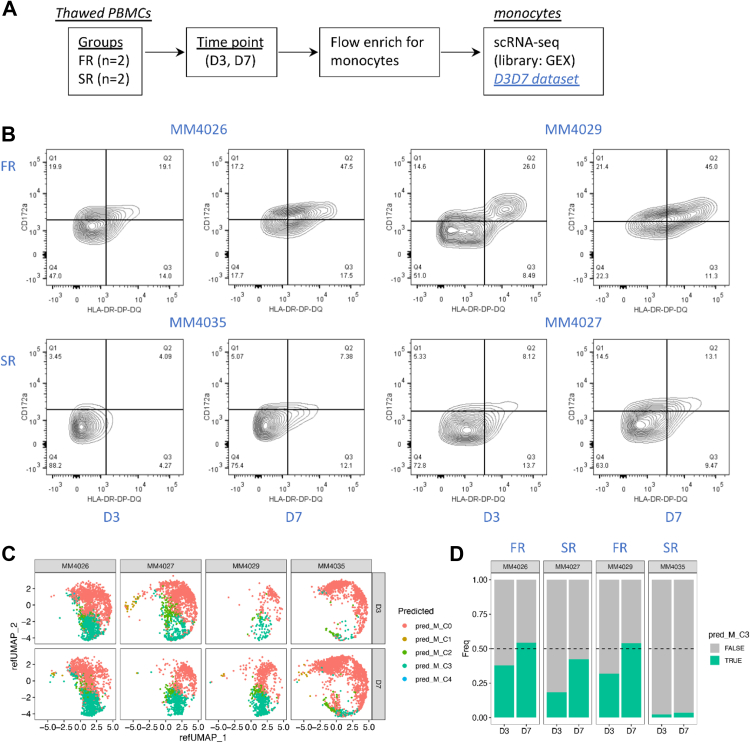
**scRNA-seq confirms the presence and further increase in M_C3 along the recovery timeline.** (A) Experimental design and workflow to generate the D3D7 dataset. (B) Flow cytometry results for CD14+ monocytes for each patient sample after thawing. Gating for CD14+ monocytes is shown in Fig. S3. (C) Using the scRNA-seq data from the DOGMA-seq dataset as a reference, the CD14+ monocytes from this dataset were predicted to be the M_C0–C6 clusters defined within the DOGMA-seq dataset and projected onto the DOGMA-seq wnnUMAP structure. (D) Composition of predicted cell clusters shown by patients at two different time points.

Because the M_C3 cell cluster was dominant in the Patients with FR, we postulated these cells are part of a resolution response and if true, their abundance in the circulation would increase over time. To test this hypothesis, cells from patients with trauma were analysed at day 3 (D3) and day 7 (D7) after trauma (Fig. 3A, Table S1). We performed flow cytometry on four patients (two patients with FR vs. two patients with SR). After gating for CD11b+CD14+ cells (Fig. S3A), CD172a hi/HLA-II hi cells increased in frequency from 19 to 28% from D3 to D7 in the two patients with FR. In contrast, CD172a hi/HLA-II hi cells from the patients with SR, which were already at a lower level than the Patients with FR at D3, increased only slightly (3–5%) from D3 to D7 (Fig. 3B). To further confirm the expansion of M_C3 monocytes at the gene expression (GEX) level, we enriched for monocytes from PBMC from the same four patients using flow sorting by deleting T and B lymphocytes (CD3+, CD19+) and subjected these cells to scRNA-seq (named D3D7 dataset). We used the scRNA-seq data from the DOGMA-seq dataset as a reference to annotate the CD14+ monocytes generated in this D3D7 dataset (Fig. 3C). The composition of the cells predicted to be M_C3 (pred_M_C3) were roughly consistent with the flow cytometry findings displaying a higher percentage in patients with FR, and with increases along the recovery timeline (Fig. 3D). It is notable that cells predicted to be M_C1 (pred_M_C1, HC-related) were very low in number in all four patients with trauma at both time points regardless of FR or SR status (Fig. 3C). This indicates that the circulating CD14+ monocytes in patients, even if in the patients with FR that had recovered from critical illness, were very different from the baseline state.

In this study, we demonstrated that the CD172a hi/MHCII hi monocytes identified through flow cytometry generally corresponded to the M_C3 monocytes we described using DOGMA-seq. To statistically quantify the dynamics of CD172a hi/MHCII hi monocytes across different recovery trajectories, we conducted flow cytometry on additional samples from two patients with FR and two patients with SR at the D3 and D7 time points (Fig. S3B). We then performed a paired t-test to compare the two time points within each recovery trajectory (Fig. S3C–D). In the patients with FR, the p-value was 0.0068, indicating a significant increase in CD172a hi/MHCII hi monocytes from D3 to D7. In contrast, there was no significant difference (p = 0.92, paired t test) observed in the patients with SR. These results suggest that the emergence of CD172a hi/MHCII hi monocytes (M_C3) is associated with a favourable recovery trajectory.

It is also notable that the changes in CD14+ monocytes (both protein markers and gene expression) followed a pattern of continuous transition rather than distinct subsets that can be clearly separated by the two surface markers. Thus, we turned our emphasis to characterising the transcriptomic changes that align with the recovery timeline to seek gene expression signatures and biological insights.

### Gene co-expression network analysis identifies a network transition (red module to green module) associated with the recovery timeline

**Fig. 4:**
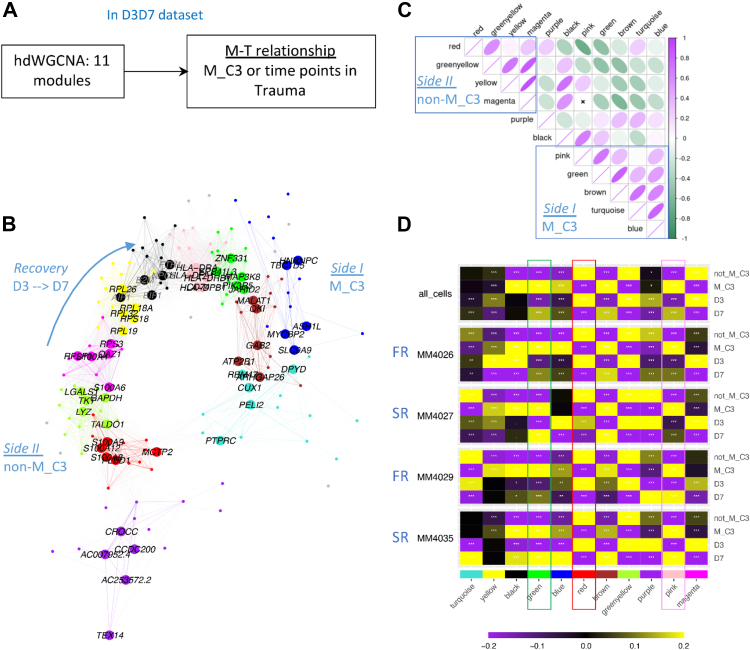
**Gene co-expression network analysis identifies red-to-green network transition associated with the recovery timeline in D3D7 dataset.** (A) Illustration of analytic workflow. (B) Visualisation of all identified networks. The top 5 hub genes for each module is shown. (C) Visualisation of the correlation between each module. (D) Module-trait relationship computed in D3D7 dataset. The colour gradient legend represents correlation coefficient.

Genes that are highly co-expressed are typically involved in the same key biological process.^20^ To identify the gene co-expression networks (modules) in the D3D7 dataset, we used the hdWGCNA package^17^ (schematic shown in Fig. 4A). As shown in Figs. 4B, 11 gene co-expression modules labelled by colours (Supplemental spreadsheet 1) were identified across all cell clusters. Generally, the modules that were close to each other in the network map had the highest positive inter-network correlation, while the modules far from each other had minimal or negative correlation. The extent of the correlation between each pair of gene modules is displayed by the correlation matrix in Fig. 4C. The modules largely fell into two groups, aligning on the two ends along the transition landscape. As shown in the module-trait relationship (Fig. 4D), side (I) (blue, brown, green modules) was dominant in the resolution-associated M_C3 cells, while the side (II) modules (red and yellowgreen) were dominant in the non-M_C3 cells. This network transition also aligned well with the shift associated with recovery from D3 to D7.

**Fig. 5:**
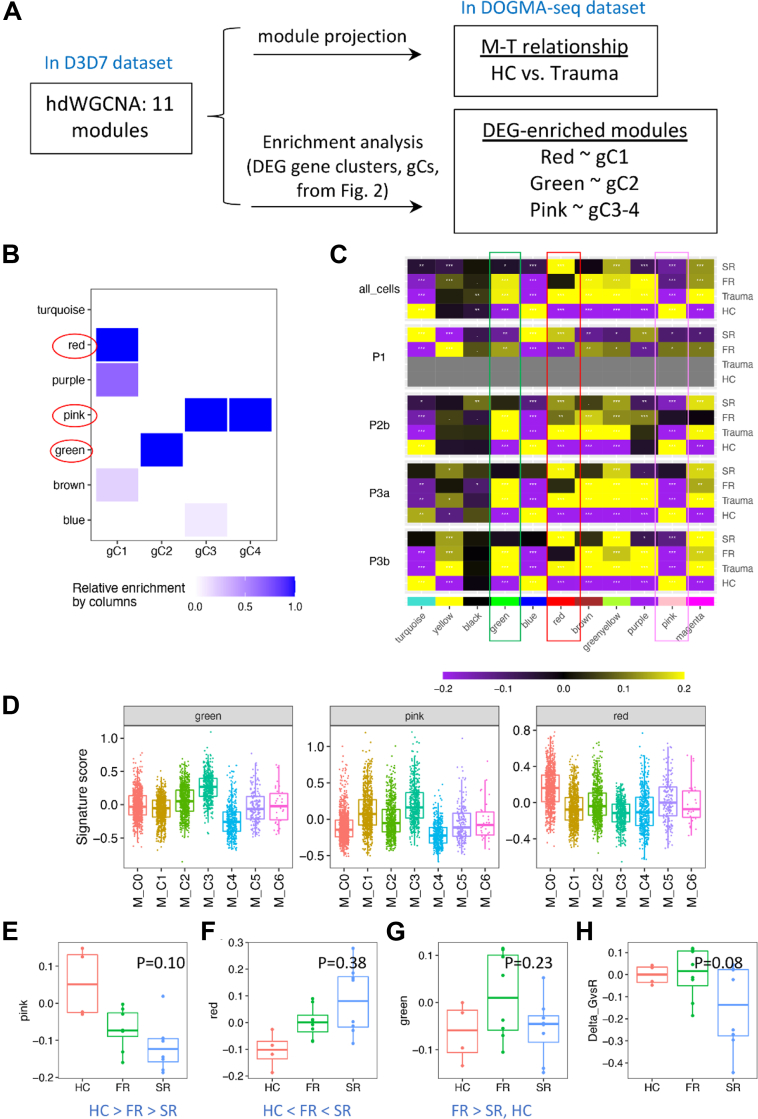
**Validation of the gene co-expression network in DOGMA-seq dataset.** (A) Illustration of analytic workflow. (B) Fold enrichment was computed between modules and gene clusters (gC) identified in Fig. 2E and then scaled between 0 and 1 for each gene cluster. (C) Modules identified in Fig. 4 were projected onto the DOGMA-seq dataset. The heatmap displays module-trait relationships, with the colour gradient representing the correlation coefficient. (D) Signature were calculated in each single cell and colour coded by cell clusters. Each dot represents a single cell. (E–H) Signature scores were averaged in each individual and colour coded by patient recovery trajectory. Each dot represents an individual. Wilcoxon test p value was computed by comparing FR and SR. The boxes shown in boxplot span from the Q1 to the Q3, with the centreline showing the median. Lower whiskers represent Q1 − 1.5∗IQR, and upper whiskers represent Q3 + 1.5∗IQR (Q1: the first quantile, Q3: the third quantile, IQR = Q3 − Q1).

Because gene modules were identified based on correlation, which uses methods distinct from DEG identification, the modules most enriched in DEG should be the most important. As such, we performed gene set enrichment analysis and identified the highest enriched gene module for each gene cluster (gC, shown in Fig. 2E). This highlighted three modules (red, green, pink) corresponding to gC1, gC2, gC3–4, respectively (Fig. 5A–B).

Next, we projected the networks identified from the D3D7 dataset to the initial DOGMA-seq dataset, to explore the module association with patients with trauma vs. HC (Fig. 5C). Three of the modules on side (I) (turquoise, blue, pink) were dominant in HC. The pink module is highly enriched in DEGs from gC3 and gC4 (Fig. 5B) and included many HLA-II genes. The under-representation of the pink module in trauma was consistent with published results showing that HLA-II genes are dramatically suppressed after injury.^2^^,^^3^^,^^7^ The remainder of the modules in the networks were induced after trauma, including another two modules highly enriched in DEGs (red and green, Fig. 5B). An important distinction between these two modules was that the green module was dominant in patients with FR, while the red module was dominant in Patients with SR, as shown in the module-trait (M-T) relationship in Fig. 5C. This association of the red and green modules with recovery rates was highly consistent across different experimental batches. The red module was enriched in the DEG cluster gC1, the DEGs upregulated across all trauma-related clusters. In contrast, the green module was enriched in gC2 that were specific to the DEG in FR-specific cell cluster, M_C3 (Figs. 2E, 5B and D). The enriched pathways for gC1 and gC2 are shown in Fig. 2I–J. The major features of the three DEG-enriched modules are summarised in Table 3. Both red and pink modules represent the trauma induced deviations in monocytes that gradually return to the baseline state. These two modules moved in opposite directions after injury; pink (HLA class II genes) suppressed (Fig. 5E) and red (pro-inflammatory genes) up-regulated (Fig. 5F), and this pattern is known to persist in the patients that recovered slowly.^3^ In contrast, the green module is a trauma-induced feature that was more pronounced in patients with early resolution of their critical illness (Fig. 5G), and thus, adds a previously undescribed monocyte trajectory that is specific to a clinical outcome.

**Table 3:** Summary of the three DEG-enriched modules.

	Red module	Pink module	Green module
Expression level	HC < FR < SR	HC > FR > SR	FR > SR, HC
Induced or suppressed by trauma	Induced	Suppressed	Induced
Return to baseline	Yes	Yes	No

Since both red and green modules are induced after trauma but differ based on clinical trajectory, we calculated the differences between the signature scores of the two modules. This calculation (green minus red) yielded the Delta_GvsR signature score, which was used to quantify the relative shift in circulating monocytes towards those expressing the green module after trauma (the degree of the red-to-green network transition). Therefore, a higher Delta_GvsR signature score represents a more green-shifted pattern along the network transition, and should correlate with early recovery. The lower the score, the more red-shifted and association with persistence of proinflammatory monocytes with suppressed HLA-class II. As expected, these patterns were evident in the DOGMA-seq dataset with patients with SR more red-shifted and FR more green-shifted at the 72 h timepoint (Fig. 5H).

### Analysis of an external trauma dataset validates the clinical association of the red-to-green network transition

To further confirm the clinical outcomes association of the red-to-green network transition, we queried a published bulk gene microarray dataset of whole blood leukocytes from 167 patients with trauma. The patients were observed up to 28 days after the trauma occurrence. Using this dataset, we previously identified two independent sources of trauma patient heterogeneity; one based on transcriptomic assessment (Signature Group [SG]) and the other based on epigenetic assessment (Epigenetic Group [EG]),^7^^,^^8^ which were independently associated with differential recovery rates. We calculated Delta_GvsR signature score for all the samples (each patient at each specific time point). The time-to-event analysis was performed at each time point, with that corresponding time point as the start date. The censoring rate for each time point was calculated as shown in Table S2.

**Fig. 6:**
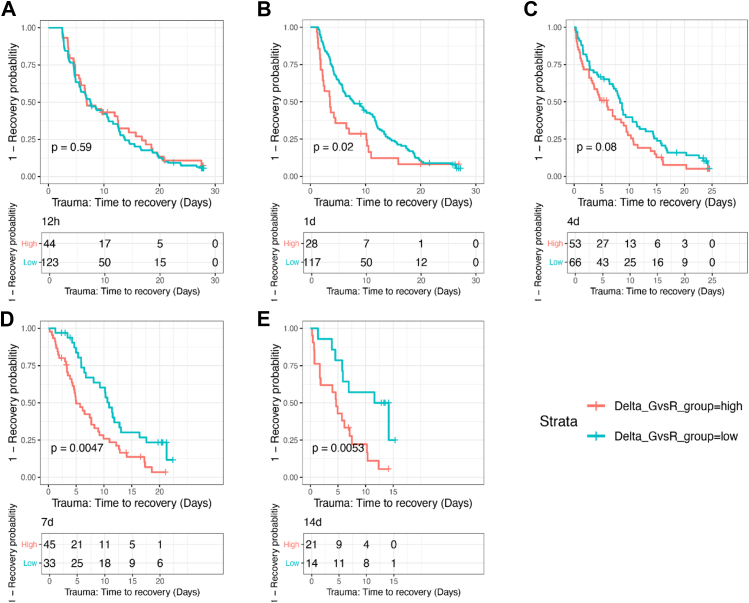
**The clinical association of the red-to-green network transition was validated in an external trauma dataset.** Dataset: GSE36809. Kaplan–Meier curves show time-to-event analysis (event = recovery from organ dysfunction) at (A) 12 h, (B) 1 day, (C) 4 days, (D) 7 days, and (E) 14 days. Analysis includes only data points prior to patient recovery. Corresponding raw p-values are indicated.

We first performed univariate time-to-event (event = recovery) analysis at sequential time points following injury (<12 h, 1 d, 4 d, 7 d, and 14 d). We calculated the median across all the data points as a cutoff. Then, the patients were classified into high or low groups depending on whether the signature score is higher or lower than the median. The Kaplan-Meier plots for each analysed time point with corresponding log-rank p value were shown in Fig. 6. After that, the Delta_GvsR signature score was also evaluated using Cox proportional hazards regression models to assess its association with recovery. Models were adjusted for major covariates including age, sex, injury severity score, neutrophil count estimated by CIBERSORT,^19^ and previously defined patient SG and EG subtypes. The results are summarised in Table 4.

**Table 4:** Multivariable Cox Proportional Hazards Analysis of Delta_GvsR signature at different time points after trauma.

Time Point	N	Hazard Ratio (HR)	95% CI	p-value
12 h	167	1.31	0.68–2.50	0.406
1 d	145	2.74	1.71–4.96	0.017
4 d	119	1.78	0.89–3.50	0.089
7 d	78	3.70	1.33–10.40	0.013
14 d	35	3.66	1.23–11.00	0.020

As shown before,^7^^,^^8^ the SG and EG subtypes assessed at <12 h after injury were significantly associated with differential recovery, while the Delta_GvsR signature score was not evident at this early timepoint. However, a higher Delta_GvsR signature score began to be associated with faster recovery around day 1 after injury and this trend remained significant at multiple later time points (Fig. 6, Table 4). Thus, we were able to validate the association between the red-to-green network transition and a faster recovery trajectory from critical illness in a large cohort of patients with trauma after adjusting for previously established prognostic factors.

### The green and red modules are associated with distinct mechanisms of regulation

**Fig. 7:**
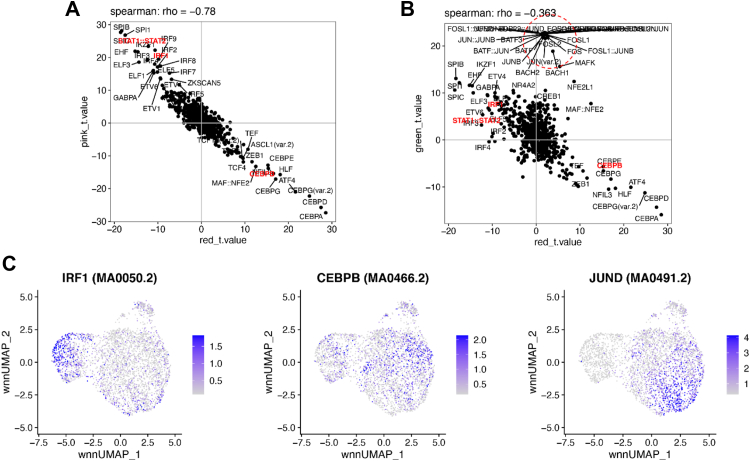
**The green and red modules are associated with different open chromatin patterns.** ChromVAR motif activity scores were computed using scATAC-seq data from DOGMA-seq dataset. Gene module signature scores were computed using scRNA-seq data from DOGMA-seq dataset. For a specific gene module and a specific motif, linear regression was fitted between signature scores and motif activity scores. (A) Comparison of t values between pink and red modules. Representative motifs are labelled in red and bold. (B) Comparison of t values between green and red modules. (C) Visualisation of motif activity scores in wnnUMAP plot as shown in Fig. 2.

To explore the underlying regulatory mechanisms associated with the three major gene co-expression modules (pink, red, green), we performed linear regression between each gene module signature score (computed using scRNA-seq) and ChromVAR motif score (computed using scATAC-seq) in our DOGMA-seq dataset. As shown in Fig. 5E and F, both the red and pink modules contain features that transition towards the baseline over time (Red: SR > FR > HC; Pink: SR < FR < HC). The regulation of the red module and the pink module was highly inversely correlated (Fig. 7A). For example, IRF1, STAT1, and STAT2 are transcription factors for IFN signalling,^21^ which are suppressed after trauma. The high accessibility of these motifs was associated with high levels of the pink module and low levels of the red module. CEBPB is a transcription factor known to be essential for emergency myelopoiesis,^22^ and its high accessibility was associated with low levels of the pink module and high levels of the red module.

In contrast, the green module represents a trauma-induced gene network that does not transition towards the baseline state (Fig. 5G). This module was distinctly associated with high availability of many AP1 family motifs, and these minimally correlated with the red and pink modules (Fig. 7B–C). Thus, although red and green modules were both stimulated after trauma and contained genes involved in inflammatory pathways, the underlying mechanisms regulating gene expression were specific to the co-expression module. Transcription factors in the AP1 family are known to promote monocyte differentiation to macrophages,^23^ but have little impact on neutrophil inflammatory responses.^24^ This led us to explore potential associations between neutrophils/monocytes and red/green module.

We next looked at the immune cell composition and the signature score for each module within the large-scale bulk transcriptomic trauma data^3^ mentioned above (Fig. S4A). Neutrophil expansion was mildly associated with the higher level of the red module (Spearman: ρ = 0.246, p = 4.75∗10ˆ−5) rather than the green module (Spearman: ρ = −0.073, p = 0.0419). In contrast, an increase in monocyte composition was mildly associated with the level of the green module gene signature (Spearman: ρ = 0.155, p = 1.41∗10ˆ−5) in contrast to the red module gene signature (Spearman: ρ = 0.059, p = 0.1). Using our previously published scRNA-seq dataset of mouse bone marrow following hemorrhagic shock with tissue trauma,^7^ we found that the red module was upregulated after trauma in both neutrophil and monocyte lineages along the trajectory of myelopoiesis, and this was more prominent in the neutrophil lineage (Fig. S4B). In line with this finding, the red module contains S100A8/A9 that is known to be highly expressed in neutrophils.^25^ In contrast, the green module was only upregulated in the monocyte lineage (Fig. S4B). These results indicate that the red-to-green network transition observed in monocytes correlates with a dampening of neutrophil expansion and a shift to a more monocyte-macrophage specific response.

### The green and red modules are associated with different ADT profiles

DOGMA-seq allows us to provide a comprehensive overview of the surface markers related to the red and green modules. To provide a complete antibody-derived tag (ADT) profile related to the two modules, we performed linear regression analysis to investigate the relationship between the signature score of a specific module and surface protein expression (Fig. S4C). CD11b (highlighted in blue) served as a positive control, which is a marker for inflammatory monocytes. As expected, CD11b is significantly positively associated with the red module, while it shows no significant association with the green module. CD38 is the highest-ranked surface marker associated with the red module and has already been linked to inflammation in monocytes and macrophages.^26^ In line with the differential ADT analysis between clusters (Fig. 2G), the green module is significantly positively associated with HLA-II and CD172a, both of which are negatively associated with the red module. This analysis identifies additional top ADTs associated with the red or green modules, respectively. These surface protein candidates could be further developed into a prognostic flow panel to monitor dynamic changes in monocyte responses in patients with trauma, which could be more cost-effective than employing a single-cell assay.

### The red-to-green network transition favours a response to M-CSF stimulation in patients with trauma

We next sought to assess the association between red-to-green network transition and macrophage differentiation in patients with trauma. To achieve this, we compared two different time points (D3 vs. D7) within the same patient using three different patients as replicates.

**Fig. 8:**
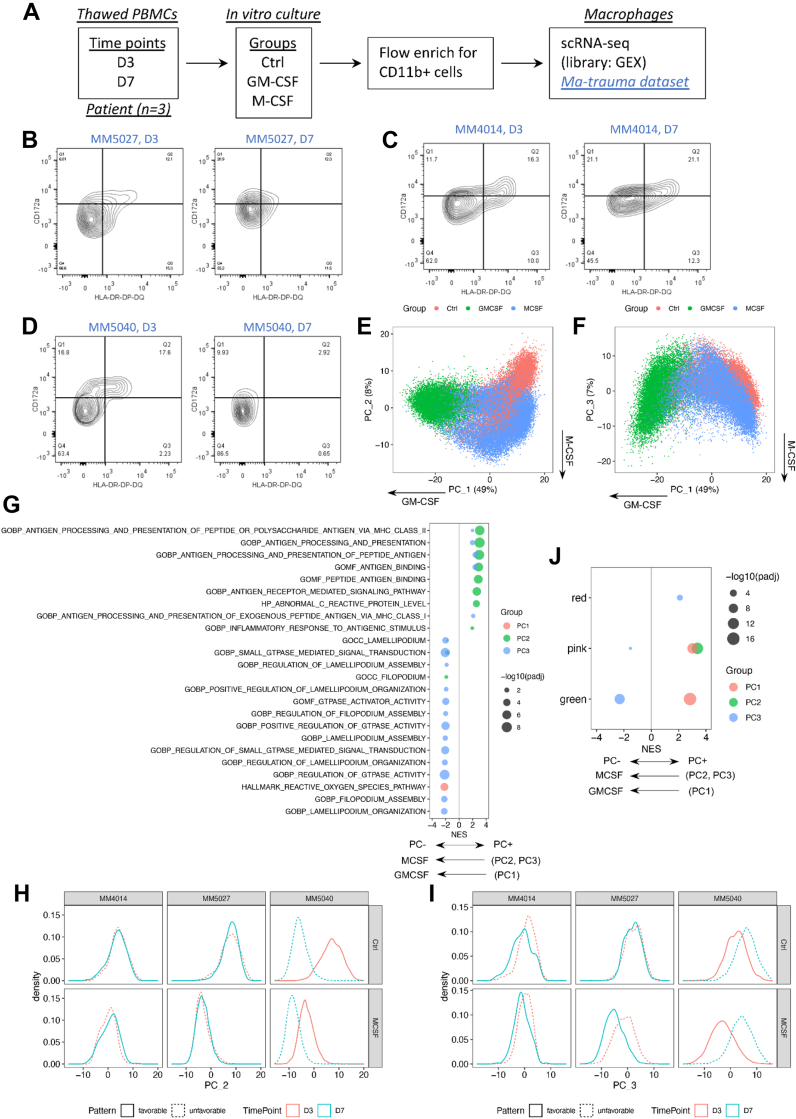
**Red-to-green network transition favours a response to M-CSF stimulation in patients with trauma.** (A) Experimental design and workflow to generate the Ma-trauma dataset. (B–D) Flow cytometry results of CD14+ monocytes for each patient sample after thawing. Gating for CD14+ monocytes is shown in Fig. S3A. (E–F) PCA plot showing the PCs representing GM-CSF (PC1) vs. M-CSF (PC2, PC3) induced changes. Each dot represents a single cell. (G) GSEA results for PC-associated genes (adjusted p-value < 0.05). Representative GO or Hallmark gene sets enriched in PC1, PC2, or PC3 are distinguished by colour. (H) Density plot for PC2. (I) Density plot for PC3. (J) GSEA results for PC-associated genes (adjusted p-value < 0.05). Red, green and pink modules enriched in PC1, PC2, or PC3 are distinguished by colour.

PBMCs from D3 and D7 from the same patient were cultured under the following conditions: medium alone and medium with M-CSF or GM-CSF. All samples from the same patient were processed in parallel (Fig. 8A, Table S3). Both M-CSF and GM-CSF are well-known to induce macrophage differentiation. M-CSF is constitutively expressed under homoeostatic conditions and leads to a default macrophage differentiation. GM-CSF is low in homoeostatic conditions, but is quickly elevated during inflammation shifting macrophages in a pro-inflammatory direction.^27^ Due to the limited number of cells, MM4014 only had Ctrl and M-CSF conditions. After 7 days, the cultured PBMCs were flow sorted for CD11b+ cells (MM5027, MM5040) or to remove dead cells (MM4014) and subjected to scRNA-seq. Flow cytometry was also performed on an aliquot of baseline samples after thawing to establish the baseline state prior to cell culture. For participant MM5027 (ICU LOS = 13, Fig. 8B) and MM4014 (ICU LOS = 22, Fig. 8C), the percentage of CD172a+ cells showed an increase at D7, indicative of a trend towards recovery from D3 to D7. In MM5040 (ICU LOS = 37, Fig. 8D), there was an obvious drop in the percentage of cells in the CD172a hi/HLAII hi quadrant between D3 and D7, suggesting a worsening of the patient's immune dysfunction at D7 compared to D3. As such, for each patient, the sample with a higher ratio of CD172a hi/HLA-II hi was labelled as favourable, and the other sample as unfavourable.

The scRNA-seq dataset generated from this experiment is referred as Ma-Trauma. To establish the changes among different experimental groups, we performed principal component analysis (PCA) (Fig. 8E–F, Fig. S5A–B). Each principal component (PC) captures a distinct pattern of variation, and different PCs are uncorrelated with each other by definition.^28^ We identified separate principal components (PC) for the GM-CSF (PC1) and M-CSF (PC2 and PC3) induced changes. Thus, the corresponding PC coordinates could be used as a quantitative parameter for the magnitude of changes induced by each cytokine. The PC1-correlated genes were mainly related to the reactive oxygen species (ROS) pathway (Fig. 8G). PC2 genes were associated with MHC-II and antigen presentation, and PC3 genes were enriched in pathways associated with cytoskeleton and motility (e.g., actin, GTPase, endocytosis, filopodia and lamellipodia) (Fig. 8G). GM-CSF induced a shift to PC1-, indicating an increase in ROS pathways. M-CSF induced a shift to PC2- and PC3-, associated with suppressing MHC-II signalling while promoting cell motility and wound healing. This analysis recapitulated the well-established roles of GM-CSF and M-CSF. Furthermore, it also showed that M-CSF induced decrease in MHC-II molecules and increase in pathways related to wound healing are relatively two independent programs that aligned on two different PCs.

The samples labelled as favourable or unfavourable were assessed for differences after cytokine stimulation, using the corresponding PC coordinates as a quantitative parameter for the magnitude of changes. GM–CSF–induced inconsistent (from PC1- to PC1+) between D3 and D7 across different patients (Fig. S5C). However, there were consistent separations of M-CSF induced changes in PC2 and PC3 (Fig. 8F–H). So we mainly focused on these two PCs.

For PC2, M-CSF induced the shift to PC2- compared with Ctrl (Fig. 8E), while favourable samples mildly shifted to PC2+ compared with corresponding unfavourable samples. This was consistent with published results that M-CSF suppressed HLA-II expression^29^ and less suppression of HLA-II is associated with better outcomes.^3^ Consistently, MM5040 (ICU LOS = 37), the patient with worst outcome had the lowest HLA-II expression in the D7 sample, aligning with the most negative end of PC2 in contrast to other samples. The differences between D3 and D7 in MM5040 shrank after M-CSF treatment (Fig. 8H). This indicates that the PC2 was driven more by the intrinsic immune states of different samples rather than M-CSF treatment. PC2 represents a clinical axis of immunosuppression—specifically the loss of HLA-II expression—where different immune states of samples are the dominant driver over M-CSF treatment. For PC3, M-CSF induced the shift to PC3- compared with Ctrl and favourable samples also shifted to PC3- compared with corresponding unfavourable samples (Fig. 8I). This observation indicates that M-CSF induced changes in PC3 aligned with favourable clinical outcomes.

We next explored the relationship between the red-to-green network transition and M-CSF induced transcriptional changes (Fig. 8J). GSEA was performed using the three modules (red, green, and pink) as gene sets and genes ranked by their correlation with a specific PC. We found that PC2+ genes were highly enriched in the pink module, with no significant enrichment in the red or green modules. In contrast, PC3 showed significant associations with all three modules. Specifically, the red module was enriched among PC3+ genes, whereas the green and pink modules were enriched among PC3- genes. These results indicate that PC3 rather than PC2 captures the red-to-green transcriptional network transition.

Consistent with this interpretation, favourable samples were modestly shifted toward PC3- compared with unfavourable samples within each patient under the Ctrl condition. This shift became more pronounced following M-CSF treatment (Fig. 8I). To quantify this difference, we applied a linear model with patient included as a fixed effect and analysed the Ctrl and M-CSF groups separately. In both groups, the differences between favourable and unfavourable samples were highly significant (Table S4). Notably, the coefficient associated with PC3 increased from 2.02 in Ctrl to 5.73 in the M-CSF group, indicating a larger separation between favourable and unfavourable samples after M-CSF treatment.

Together, these results suggest that the red-to-green network transition represents a biologically meaningful axis of monocyte variation. This axis distinguishes favourable from unfavourable samples at baseline and is also closely involved in the beneficial transcriptional changes induced by M-CSF (increased cell motility and wound healing). The increased separation along PC3 after treatment suggests that M-CSF may amplify pre-existing transcriptional differences along the red-to-green network transition.

### Red-to-green network transition is not closely involved in M-CSF induced wound healing process in healthy participants

**Fig. 9:**
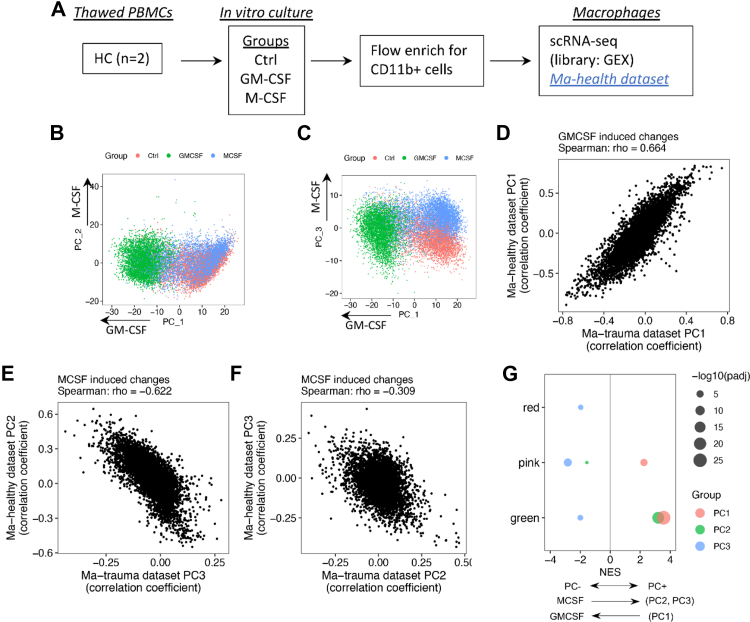
**Red-to-green network transition is not closely involved in M-CSF induced wound healing process in healthy controls.** (A) Experimental design and workflow to generate the Ma-healthy dataset. (B–C) PCA plot showing the PCs representing GM-CSF (PC1) vs. M-CSF (PC2, PC3) induced changes. Each dot represents a single cell. (D) Comparison of GM-CSF induced changes between Ma-healthy and Ma-trauma datasets. (E–F) Comparison of M-CSF induced changes between Ma-healthy and Ma-trauma datasets. (G) GSEA results for PC-associated genes (adjusted p-value < 0.05). Red, green and pink modules enriched in PC1, PC2, or PC3 are distinguished by colour.

Because both red and green modules are trauma induced, we assessed whether the cells isolated from patients with trauma respond differently from those isolated from HC. Cells from two HC were cultured with or without GM-CSF or M-CSF, and subjected to scRNA-seq (Ma-healthy dataset) (Fig. 9A, Table S3). Using the same analytic strategy, we identified the PCs associated with the GM-CSF (large shift to PC1−) or M-CSF (slight shift to PC2+ and large shift to PC3+) -induced changes (Fig. 9B–C). The Spearman correlation coefficients were calculated between the gene expression values and PC coordinates in the Ma-trauma and Ma-healthy datasets separately and then the correlation coefficients compared across the two datasets. For the GM–CSF–induced changes (PC1− in the Ma-trauma dataset, PC1− in the Ma-healthy dataset), the two datasets displayed a very high correlation (ρ = 0.664) (Fig. 9D), meaning that the monocytes from patients with trauma and HC participants respond to GM-CSF in a highly overlapping manner associated with the reactive oxygen species pathway. M-CSF induced changes, PC2 and PC3, in Ma-trauma datasets can be roughly mapped to the PC3 and PC2 of Ma-healthy datasets, respectively (Spearman's correlation = −0.622 and −0.309, Fig. 9E–F). These associations are consistent with M-CSF inducing MHC-II suppression and enhanced wound-healing programs in HC cells. However, neither PC2 nor PC3 in the Ma-healthy dataset fit within the red-to-green network transition (Fig. 9G).

These results suggest that, although M-CSF can suppress MHC-II signalling and promote wound healing programs in both healthy and trauma monocytes, the red-to-green network transition does not appear to play a central role in M-CSF induced changes in HC cells.

## Discussion

In the current paradigm, outcome-related immune response features appear very early after injury. These include an up-regulation of pro-inflammatory pathways and suppression of IFN signalling and antigen presentation machinery after trauma; changes that gradually return baseline. These changes largely align with damage-associated molecular patterns triggered responses in innate immune cells (i.e., CD14+ monocytes), and it is the magnitude and duration of these divergent responses that associate with the post-injury clinical course.^3^^,^^7^ However, in this study, we observed that the transcriptomic changes in CD14+ monocytes do not all return to baseline after trauma. Our multi-modal single cell analysis identified a subset of CD14+ monocytes (M_C3, CD172a hi/HLA-II hi) that emerge in the subacute time frame and are dominant in patients that go on to resolve their critical illness early. These cells possessed features that continuously deviate from CD14+ monocytes from HC. Our analysis of gene co-expression networks revealed a range of gene modules that define the baseline state, the previously described early trauma response,^3^ and the resolving monocytes we identified in this study. A gene module that defined the resolving monocytes (designated as the green module in the results) was used to demonstrate that the resolving monocytes are not only a newly described trauma induced monocyte subset that expands along the recovery timeline, but that these monocytes are primed for an enhanced and unique response to M-CSF for macrophage differentiation. These findings add a new dimension to the long-standing paradigm used to explain the adaptive and dysfunctional aspects of the human immune response to severe injury.

**Fig. 10:**
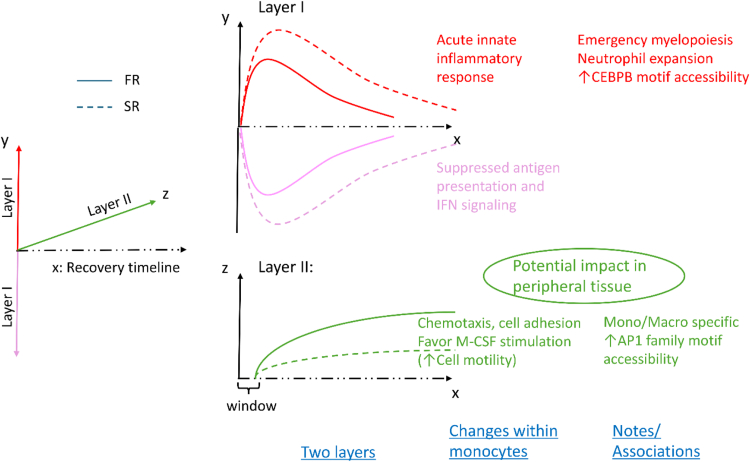
**Proposed a model with three dimensions and two layers for the immune response to trauma**.

Combining the published studies and our findings, we proposed a model with three dimensions and two layers for the immune response to trauma in the acute and subacute time frames (Fig. 10). Layer I (x vs. y) encompasses emergency myelopoiesis (neutrophil expansion), acute inflammation, and the subsequent changes in adaptive immunity (loss of lymphocytes); changes captured in the upregulation of the red module and downregulation of the pink module characterised in this study. This layer includes both trauma induced and suppressed features that gradually resolve with recovery, corresponding to the current paradigm.^3^ Layer II (x vs. z) is a new dimension characterised in this study that emerges prior to recovery and continuously increases with recovery from critical illness. This new trajectory is more specific to the monocyte-macrophage lineage and is represented by the induction of the green module. These features are associated with a modest increase in the number of circulating monocytes and favour a response to M-CSF for macrophage differentiation involving increased cell motility favourable for tissue repair. A faster recovery is associated with resolution of the changes in layer I and enhanced transition to layer II. We provide a molecular definition of this transition through the features defined by the red-to-green network transition (Delta_GvsR signature score). We note that these changes do not alter monocyte responses to GM-CSF indicating that circulating CD14+ monocytes remain capable of mounting an inflammatory response when encountering new insults.

A remarkable feature of resolving monocytes is the restoration of HLA class II surface expression. This was established at both the transcriptomic and surface protein levels. The early and dramatic loss of surface HLA is thought to be an adaptive response that prevents the indiscriminate overactivation of the immune response to antigens released from damaged tissues.^30^ However, a prolonged loss of antigen presenting capacity can render the patients more susceptible to infections as seen by the high rate of nosocomial infections seen in patients with trauma with persistent critical illness.^31^ The combination of HLA expression (restored pink module expression, layer I) with enhanced migration capacity (upregulated green module expression, layer II) suggests that these cells emerge to restore pathogen recognition and reshape the immune response in peripheral tissues.

Our DOGMA-seq analysis also provided a glance at the underlying regulatory mechanisms for the two layers. Layer I is associated with high accessibility of the CEBPB binding sites, which has already been associated with emergency myelopoiesis^22^ and we have shown is an element of the host response to trauma and shock.^7^ The Layer II monocyte response is associated with high accessibility of AP1 family binding sites, which are known to be critical in macrophage differentiation.^23^ However, which AP family member(s) are most responsible, especially in the trauma setting requires further investigation.

Multiple complementary validation approaches were described throughout the manuscript, including flow cytometry confirmation, functional *in vitro* macrophage differentiation assays, validation in a large external trauma cohort, and comparison of differential expression results with co-expression network analysis. However, our study also has limitations. First, while we provide evidence that the resolving monocytes are altered to migrate into the tissues, proof that these cells play a direct role in the recovery process will require replication of the human response in model organisms. Second, the direct analysis of cells over time was limited to D7. Details on the duration of the red-to-green shift in monocytes and whether these changes give way to other changes in monocytes post-injury would require assessing cells from later time points. Last, although patients and HCs were age- and sex-matched, the limited sample size precluded meaningful sex-stratified analyses, and race/ethnicity data were not incorporated, and the study population may not fully capture the heterogeneity of patients with trauma. Together, these factors limit the generalisability of our findings.

In summary, our in-depth single cell investigation into the early differences in circulating leukocytes based on patient recovery trajectory identified a new dimension to the long-standing paradigm for the systemic immune response to severe injury in humans. Not only does the exaggerated pro-inflammatory and counter inflammatory responses ultimately resolve to the baseline state with recovery, but a new gene co-expression pattern emerges along with recovery. The emergence of a new monocyte trajectory establishes that early resolution is defined by more than a return to baseline. The monocyte subset with a red-to-green shift in transcriptomic pattern aligns with rapid recovery and favours cell motility when exposed to M-CSF, a factor known to induce macrophage differentiation. We speculate that the shift favours tissue repair and a return of immune function. If the key regulators that shift (enhance or suppress) the red-to-green transition can be identified, it may be feasible to promote the transition of circulating monocytes to this favourable subtype. It may also be feasible to identify through drug screens using the differential transcriptomic patterns already approved drugs that can shift monocytes to the favourable gene expression patterns we have identified.

## Contributors

T.B. and T.C. conceptualised the study. T.C. performed computational analysis, cell culture and flow cytometry. J.H. prepared single-cell gene expression libraries. J.C. prepared DOGMA-seq libraries. W.C. provided the analytic pipeline for DOGMA-seq data and consulting support for the computational analyses. R.D provided the support and protocol for DOGMA-seq library preparation. T.C. and T.B. wrote the manuscript with the feedback of all the authors who have read and approved the manuscript. T.C., W.C. and T.B. have verified the underlying data.

## Data sharing statement

The data (Fastq files and processed data) have been deposited on GEO (GSE295173 and GSE295174) and in a public domain.

## Declaration of interests

All authors declare no competing interests.

## Appendix A Supplementary data

Supplementary Tables and Figures

WGCNA consensus modules
