# Supplementary material for: Multi-modal single-cell sequencing reveals network transition in circulating monocytes that aligns with faster recovery in patients with trauma and favours a response to M-CSF
Source: eBioMedicine. 2026 Jul 3;130:106368. doi: 10.1016/j.ebiom.2026.106368 (PMC13351336; doi:10.1016/j.ebiom.2026.106368)
Supplement: Supplementary Tables and Figures [file mmc1.docx]

Table S1: Sample information for D3D7 dataset.

| **Sample ID** | **Batch** | **age** | **gender** | **Injury** | **ICU days** |
| --- | --- | --- | --- | --- | --- |
| MM4026 | 1st | 42 | Male | blunt | 6 |
| MM4035 | 1st | 34 | Male | blunt | 16 |
| MM4027 | 2nd | 26 | Female | blunt | 15 |
| MM4029 | 2nd | 27 | Female | penetrating | 6 |

Table S2: Censoring rates at each time point

| **Time point** | **Censoring rate** | **Non-recovered cases (n)** | **Recovered cases (n)** |
| --- | --- | --- | --- |
| 12h | 0.089820359 | 15 | 152 |
| 1d | 0.082758621 | 12 | 133 |
| 4d | 0.117647059 | 14 | 105 |
| 7d | 0.179487179 | 14 | 64 |
| 14d | 0.228571429 | 8 | 27 |

Table S3: Sample information for Ma-trauma and Ma-healthy datasets. Groups: Ctrl, GM-CSF, M-CSF.

| **Sample ID** | **Batch** | **age** | **gender** | **Injury** | **ICU days** |
| --- | --- | --- | --- | --- | --- |
| MM5027 | 1st | 27 | Male | blunt | 13 |
| MM5040 | 2nd | 41 | Male | blunt | 37 |
| MM4014 | 5th | 63 | Male | blunt | 22 |
| HC1 | 3rd | 37 | Male | N/A | HC |
| HC2 | 4th | 33 | Male | N/A | HC |

Table S4: Fixed-effect linear model results. Models were fit separately for the Ctrl and M-CSF conditions using the formula PC3 ~ Pattern (favorable/unfavorable) + Patient, where patient identity was included as a fixed effect. Patient coefficients represent offsets relative to the reference patient (MM4014).

**Ctrl group**

| **Variable** | **Estimate** | **Std. Error** | **t value** | **p value** |
| --- | --- | --- | --- | --- |
| Intercept | -1.19782 | 0.11596 | -10.33 | <2e-16 |
| Pattern (unfavorable) | 2.02678 | 0.07844 | 25.84 | <2e-16 |
| Patient: DonorMM5027 | 2.50694 | 0.11521 | 21.76 | <2e-16 |
| Patient: DonorMM5040 | 3.97883 | 0.11439 | 34.78 | <2e-16 |

**M-CSF group**

| **Variable** | **Estimate** | **Std. Error** | **t value** | **p value** |
| --- | --- | --- | --- | --- |
| Intercept | -5.50266 | 0.06092 | -90.33 | <2e-16 |
| Pattern (unfavorable) | 5.72620 | 0.05660 | 101.17 | <2e-16 |
| Patient: DonorMM5027 | -0.58756 | 0.06145 | -9.56 | <2e-16 |
| Patient: DonorMM5040 | 3.03234 | 0.05432 | 55.82 | <2e-16 |

**SUPPLEMENTAL FIGURE AND FIGURE LEGENDS**

**
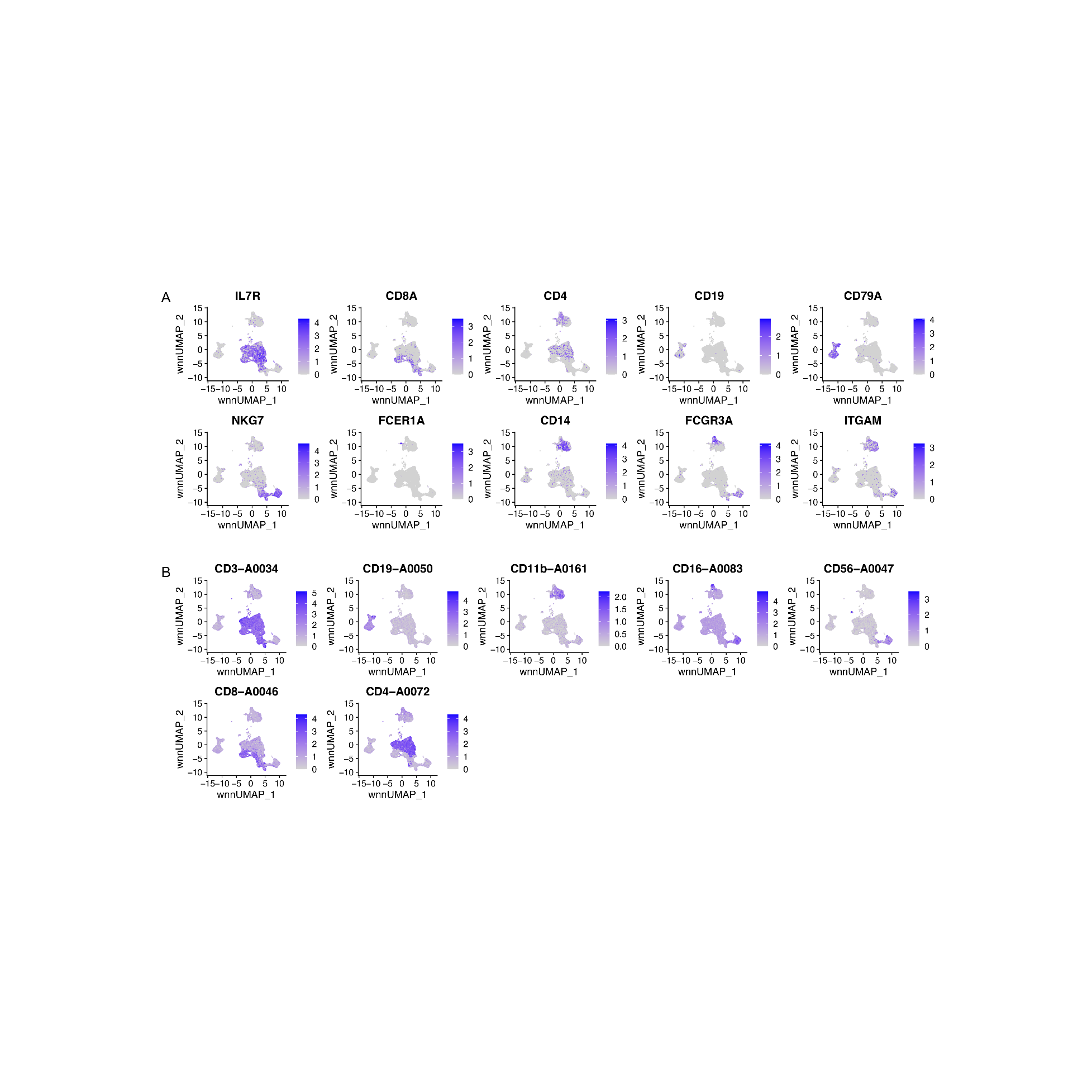
**

**Fig. S1. Expression of representative features in PBMCs from DOGMA-seq dataset** (related to Fig. 1). (A) Gene RNA expression. (B) ADT protein expression.


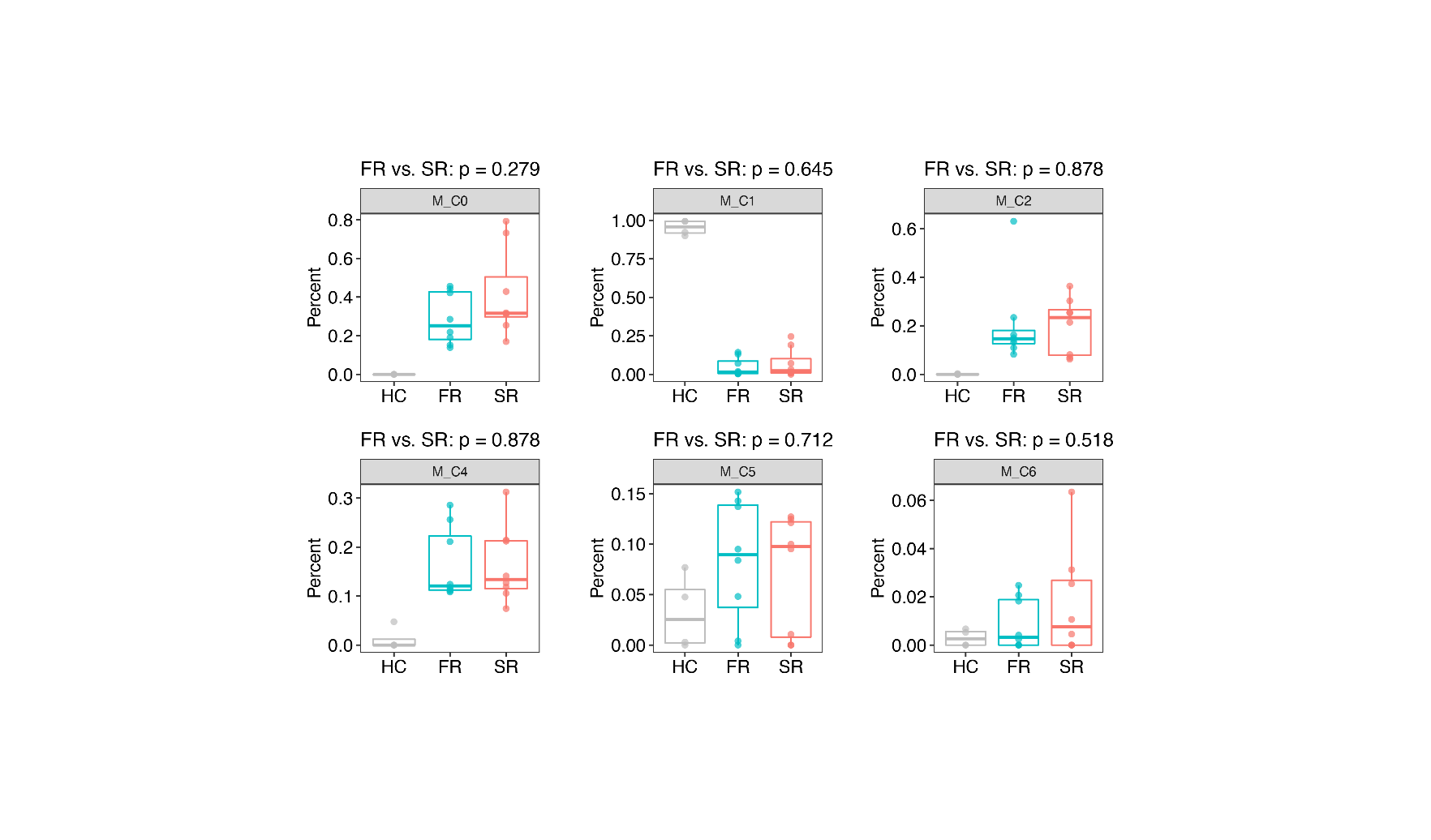


**Fig. S2. The percentage of each cluster was shown by individuals** (related to Fig. 2). Each dot represents an individual. The differences between FR and SR were tested by Wilcoxon test.


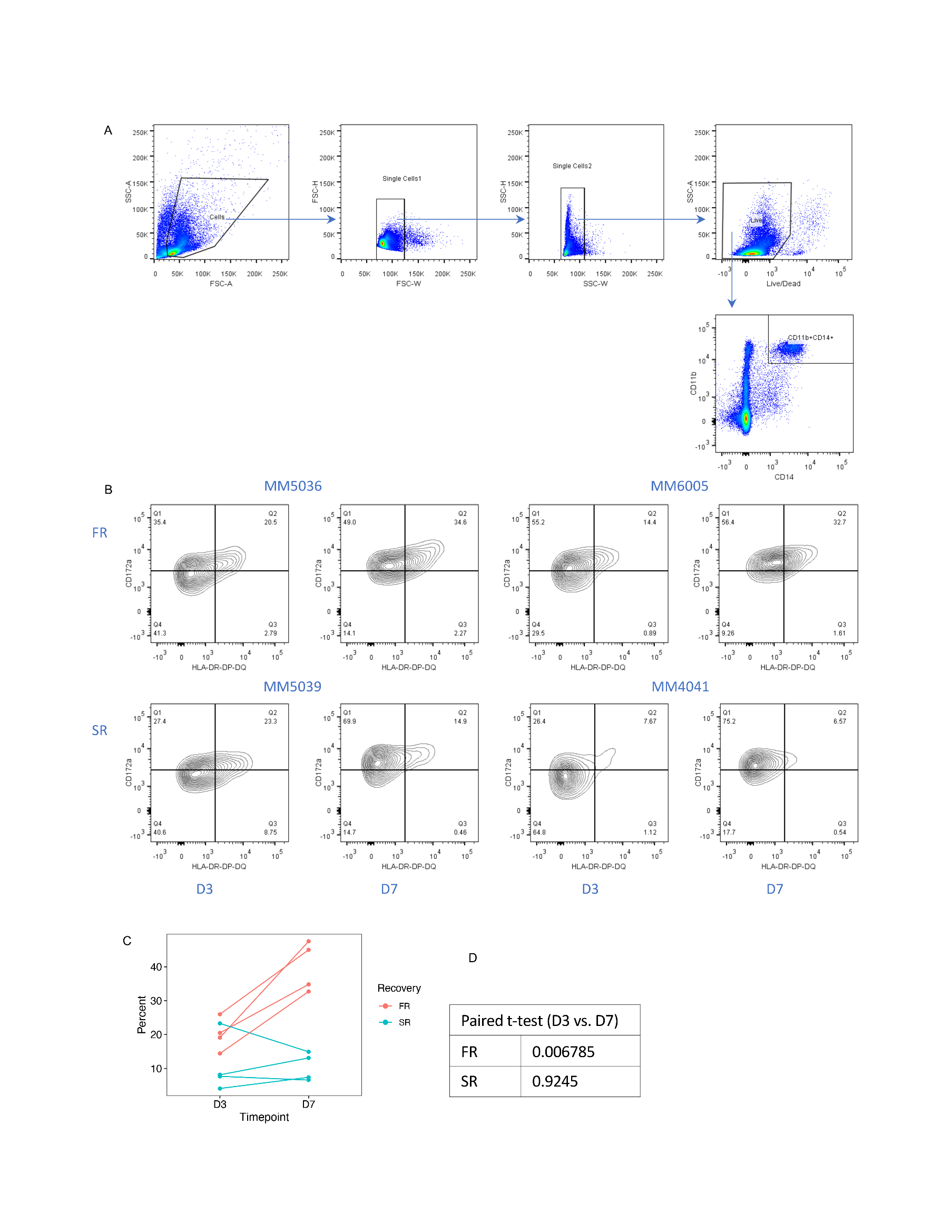


**Fig. S3. Statistical quantification of flow results** (related to Fig. 3). (A) Gating strategy for CD14+ monocytes. (B) Flow cytometry on additional samples from two FR and two SR patients at the D3 and D7 time points. (C) Percentage of CD172a hi/MHCII hi monocytes from D3 to D7 for each of the four patients shown in Fig. 3B and Fig. S3B. (D) Statistical results.


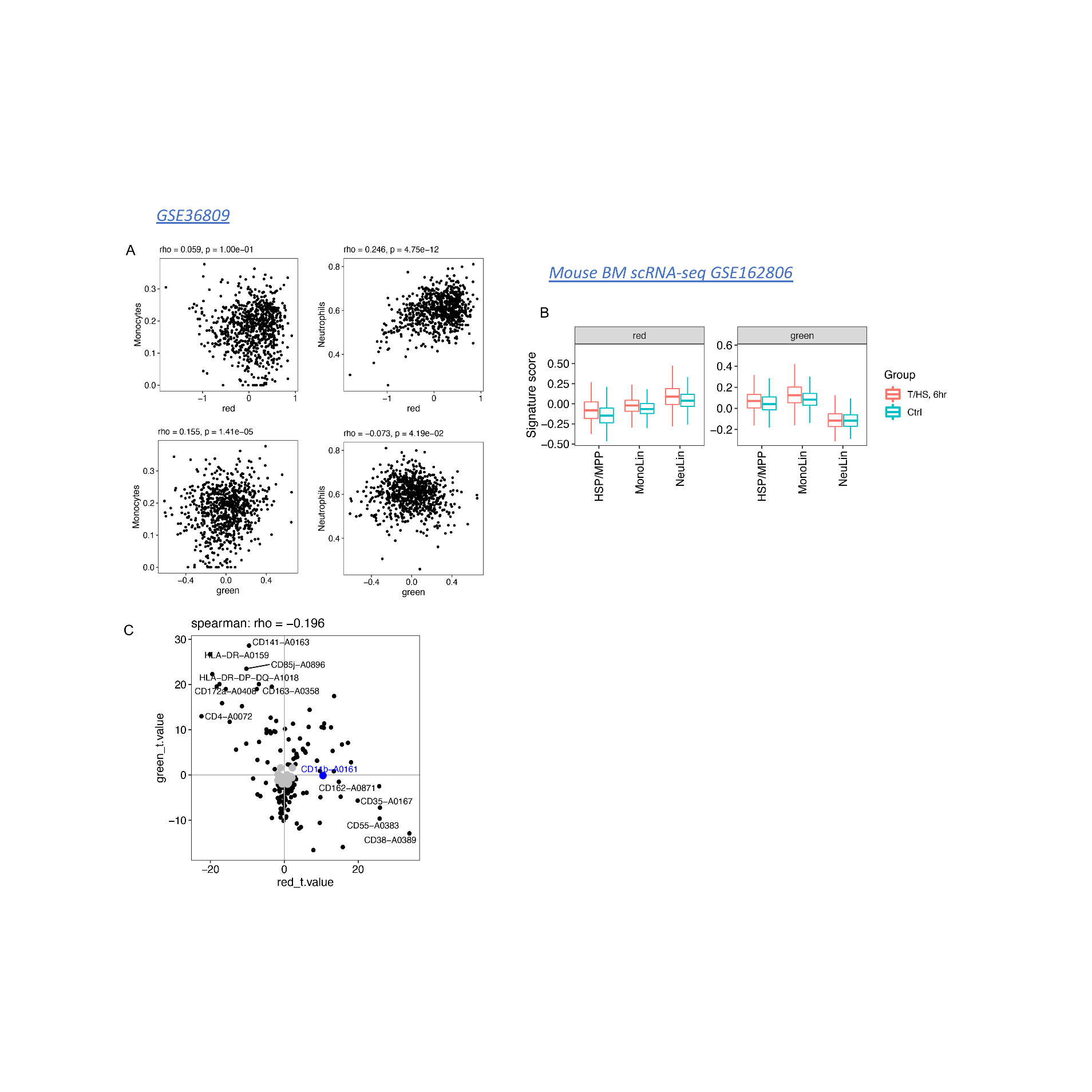


**Fig. S4. Association of gene modules with neutrophils or monocytes** (related to Fig. 7). (A) Dataset: GSE36809. Neutrophil and monocyte composition were deconvoluted by CIBERSORT. Correlations were shown between cell compositions and gene module signature scores. Each dot represents each patient. (B) Dataset: GSE162806. Gene module signature scores were calculated in the bone marrow cells in myeloid lineages 6 hours after polytrauma. (C) ADTs correlated to the red or green module. For a specific gene module and a specific ADT, linear regression was fitted between signature scores and ADT expression value. Grey dots represent ADTs with no significant association with either module.


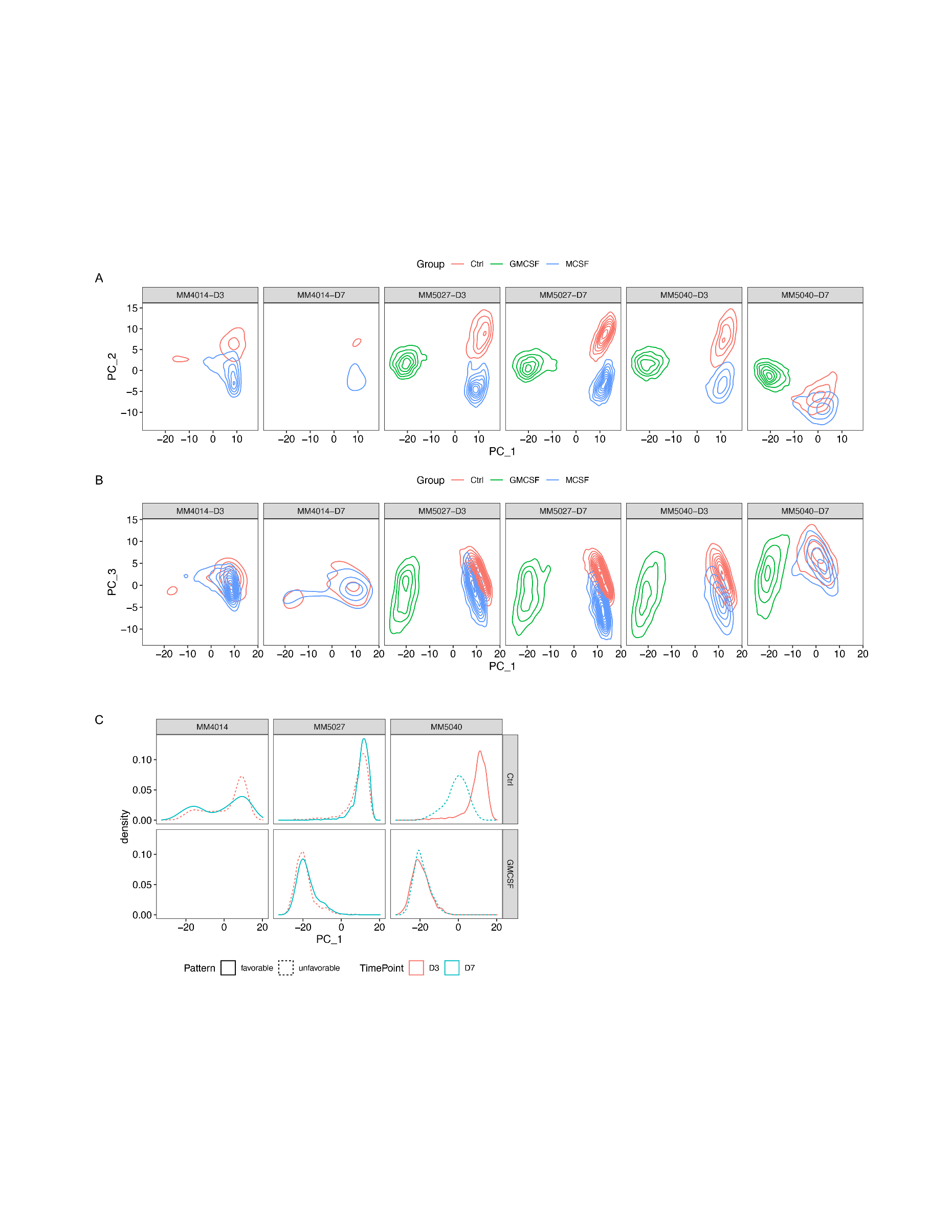


**Fig. S5. PC interpretation for Ma-trauma dataset** (related to Fig. 8). (A-B) PCA 2D-density plot shown by each sample. (A) PC1 vs PC2. (B) PC1 vs PC3. (C) Density plot for PC1 (GM-CSF induced changes).


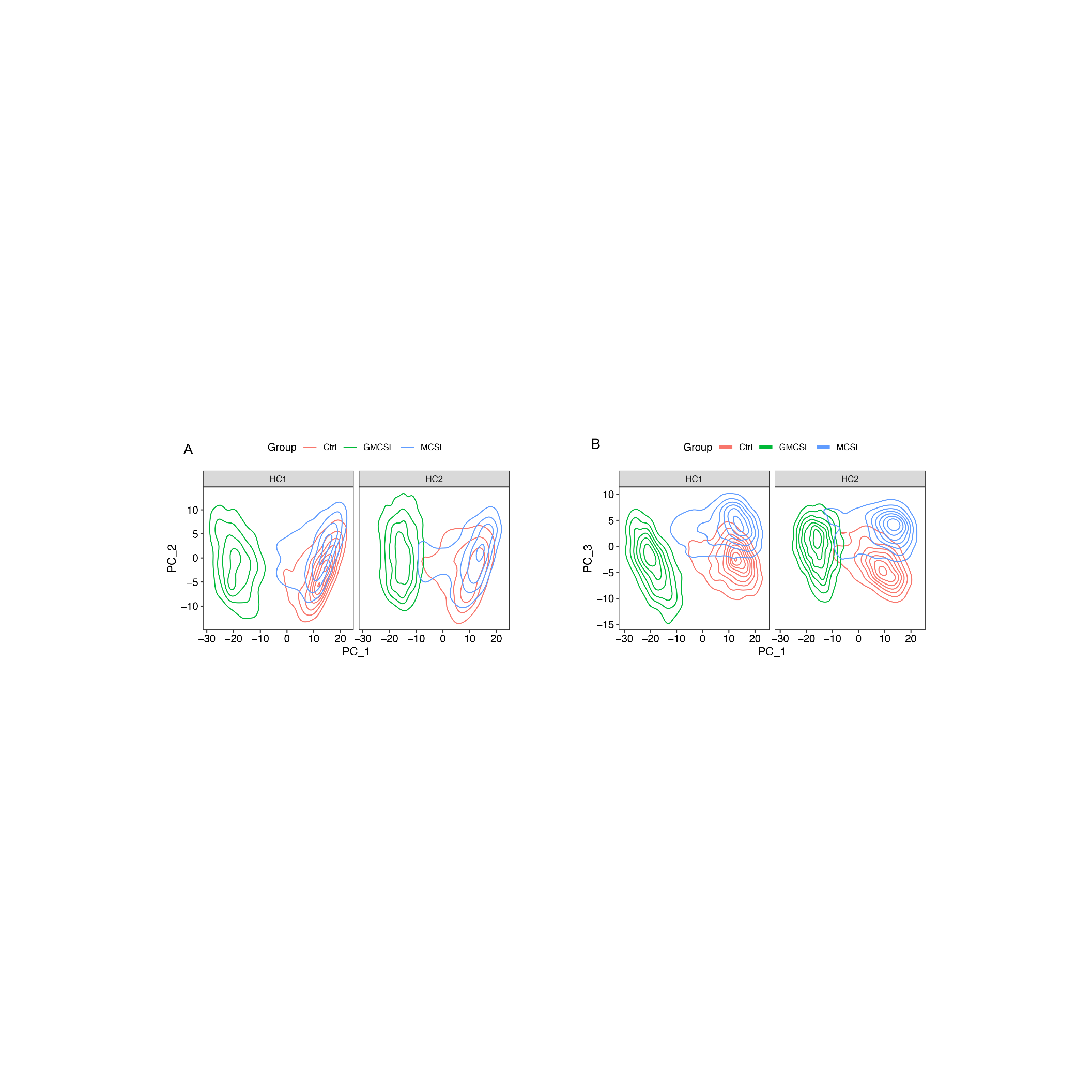


**Fig. S6. PC interpretation for Ma-healthy dataset** (related to Fig. 9). (A-B) PCA 2D-density plot shown by each sample. (A) PC1 vs PC2. (B) PC1 vs PC3.
